# Estimated Cost-effectiveness of Endoscopic Screening for Upper Gastrointestinal Tract Cancer in High-Risk Areas in China

**DOI:** 10.1001/jamanetworkopen.2021.21403

**Published:** 2021-08-17

**Authors:** Ruyi Xia, Hongmei Zeng, Wenjun Liu, Li Xie, Mingwang Shen, Peng Li, He Li, Wenqiang Wei, Wanqing Chen, Guihua Zhuang

**Affiliations:** 1 Department of Epidemiology and Biostatistics, School of Public Health, Xi’an Jiaotong University Health Science Center, Xi’an, China; 2 National Cancer Center/Cancer Hospital, Chinese Academy of Medical Sciences and Peking Union Medical College, Beijing, China

## Abstract

**Importance:**

Upper gastrointestinal tract cancer, including esophageal and gastric cancers, in China accounts for 50% of the global burden. Endoscopic screening may be associated with a decreased incidence of and mortality from upper gastrointestinal tract cancer.

**Objective:**

To evaluate the cost-effectiveness of endoscopic screening for esophageal and gastric cancers among people aged 40 to 69 years in areas of China where the risk of these cancers is high.

**Design, Setting, and Participants:**

For this economic evaluation, a Markov model was constructed for initial screening at different ages from a health care system perspective, and 5 endoscopic screening strategies with different frequencies (once per lifetime and every 10 years, 5 years, 3 years, and 2 years) were evaluated. The study was conducted between January 1, 2019, and October 31, 2020. Model parameters were estimated based on this project, government documents, and published literature. For each initial screening age (40-44, 45-49, 50-54, 55-59, 60-64, and 65-69 years), a closed cohort of 100 000 participants was assumed to enter the model and follow the alternative strategies.

**Main Outcomes and Measures:**

Cost-effectiveness was measured by calculating the incremental cost-effectiveness ratio (ICER), and the willingness-to-pay threshold was assumed to be 3 times the per capita gross domestic product in China (US $10 276). Univariate and probabilistic sensitivity analyses were conducted to assess the robustness of model findings.

**Results:**

The study included a hypothetical cohort of 100 000 individuals aged 40 to 69 years. All 5 screening strategies were associated with improved effectiveness by 1087 to 10 362 quality-adjusted life-years (QALYs) and increased costs by US $3 299 000 to $22 826 000 compared with no screening over a lifetime, leading to ICERs of US $1343 to $3035 per QALY. Screening at a higher frequency was associated with an increase in QALYs and costs; ICERs for higher frequency screening compared with the next-lower frequency screening were between US $1087 and $4511 per QALY. Screening every 2 years would be the most cost-effective strategy, with probabilities of 90% to 98% at 3 times the per capita gross domestic product of China. The model was the most sensitive to utility scores of esophageal cancer– or gastric cancer–related health states and compliance with screening.

**Conclusions and Relevance:**

The findings suggest that combined endoscopic screening for esophageal and gastric cancers may be cost-effective in areas of China where the risk of these cancers is high; screening every 2 years would be the optimal strategy. These data may be useful for development of policies targeting the prevention and control of upper gastrointestinal tract cancer in China.

## Key Points

**Question:**

Is endoscopic screening for esophageal and gastric cancers cost-effective in areas of China where the risk of these cancers is high?

**Findings:**

In this model-based economic evaluation of a hypothetical closed cohort of 100 000 individuals aged 40 to 69 years from areas of China where the risk of upper gastrointestinal tract cancer is high, 5 endoscopic screening strategies with different frequencies had an incremental cost-effectiveness ratio of less than the per capita gross domestic product in China for each quality-adjusted life-year gained compared with no screening. Screening every 2 years would be the most cost-effective strategy.

**Meaning:**

These findings suggest that combined endoscopic screening for esophageal cancer and gastric cancer would be cost-effective in areas of China where the risk of these cancers is high and that screening every 2 years would be the optimal strategy.

## Teaser

This economic evaluation assesses the cost-effectiveness of endoscopic screening for esophageal and gastric cancers in a hypothetical cohort of individuals aged 40 to 69 years in areas of China where the risk of upper gastrointestinal tract cancer is high.

## Introduction

The incidence of upper gastrointestinal tract cancer (UGIC), including esophageal cancer (EC) and gastric cancer (GC), is high in China, with an estimated 802 930 new cases (324 422 cases of EC and 478 508 cases of GC) and 674 924 deaths (301 135 due to EC and 373 789 due to GC) in 2020, accounting for approximately 50% of the global burden.^1^ However, the geographic distribution of UGIC is uneven in China, mortality rates in some areas being 2- to 3-fold higher than the national average.^2,3,4^

The prognosis of UGIC depends mainly on the disease stage at the time of diagnosis. In China, the overall 5-year survival rates for patients with EC and GC are 30.3% and 35.1%, respectively.^5^ However, the rates would be 86% and 90%, respectively, if disease were detected at an early stage.^6,7^ This potential for increased survival has provided justification for early detection programs. A series of nationwide screening programs has been established in several East Asian countries where the incidence rates are high. In Japan, radiographic screening for GC was developed in the 1960s, and a national screening program was established in 1983; currently, endoscopic screening every 2 to 3 years is usually recommended.^8^ South Korea introduced both radiographic screening and endoscopic screening for GC into national screening programs in 2000 and currently recommends endoscopic screening every 2 years.^9^ Over approximately the past 20 years, endoscopic examination has become a major screening method for UGIC because of its high accuracy.^10^ Several economic evaluation studies^11,12,13,14,15,16^ from South Korea, Singapore, Portugal, the US, and China showed that endoscopic screening for EC or GC was cost-effective compared with no screening. Another study^17^ conducted in the US suggested that the cost-effectiveness of combined endoscopic screening for EC and GC was comparable to that of funded screening programs for other cancers when it was integrated into the current colonoscopy screening program.

In China, several endoscopic screening programs for UGIC have been attempted in some areas with high incidence rates since 2005. Some observational studies^18,19,20,21,22^ have shown that endoscopic screening can reduce the incidence of and mortality associated with UGIC. To assess the feasibility and efficacy of endoscopic screening for EC and GC, a multicenter randomized controlled trial was launched in 2015, in which more than 140 000 persons aged 40 to 69 years were enrolled.^23,24^ A large amount of basic data has been obtained from this trial. The present study evaluated the cost-effectiveness of combined endoscopic screening for EC and GC in people aged 40 to 69 years in areas of China where the risk of these cancers is high. Furthermore, we evaluated the optimal initial age and frequency for screening.

## Methods

This model-based economic evaluation was performed from the health care system perspective using TreeAge Pro (Healthcare Version) 2020 (TreeAge Software) and was conducted between January 1, 2019, and October 31, 2020. The project was registered with the Protocol Registration System in the Chinese Clinical Trial Registry and approved by the independent Ethics Committee of the National Cancer Center of China/Cancer Hospital, Chinese Academy of Medical Sciences. Because patient data were deidentified in the analysis, the requirement for informed consent was waived by the Ethics Committee of the National Cancer Center of China/Cancer Hospital, Chinese Academy of Medical Sciences. This evaluation followed the Consolidated Health Economic Evaluation Reporting Standards (CHEERS) reporting guideline.^25^

### Markov Model

A Markov model was constructed for different initial screening ages (40-44, 45-49, 50-54, 55-59, 60-64, and 65-69 years) to simulate EC and GC progression and calculate related health and economic outcomes in a lifetime horizon. Five endoscopic screening strategies with different frequencies were considered, including once per lifetime and every 10 years, 5 years, 3 years, and 2 years. No screening was applied as a reference strategy. For each initial screening age, a closed cohort of 100 000 participants with a mean age of 42, 47, 52, 57, 62, or 67 years was assumed to enter the model and follow the alternative strategies.

The Markov model consisted of 26 health states. In addition to the normal and death states, series of EC and GC progression states were considered, and each progression state was divided into undetected and detected states. A posttreatment state was also considered separately from the states requiring medical treatment after diagnosis. Endoscopic screening, reexamination, and related treatment procedures followed the recommendations of the Chinese Expert Consensus on Screening and Endoscopic Diagnosis and Treatment of Early Esophageal Cancer/Gastric Cancer (2014 version).^26,27^ These recommendations were also followed in a randomized controlled trial by Chen et al.^23^ Details of the trial are given in the eMethods and eFigures 1 to 3 in the Supplement, details of the Markov model are given in eFigure 4 in the Supplement, and validation of the model is shown in eFigures 5 and 6 in the Supplement. Compliance with screening, reexamination, and treatment and complications associated with endoscopic screening were also considered in the model. The model was run with a 1-year cycle length and terminated when the mean age of the cohort reached 90 years. A half-cycle correction was applied.

### Model Parameters

Compliance with endoscopic screening was determined to be 49% (range, 30%-80%) according to a pilot project concerning endoscopic screening for EC in several areas of China where the risk of EC is high (Table 1).^18,28,29,30,31,32,33,34,35,36,37,38,39,40,41^ Compliance with regular endoscopic reexamination among individuals who had mild esophageal dysplasia, moderate esophageal dysplasia, or low-grade gastric intraepithelial neoplasia detected on screening was assumed to be higher than compliance with endoscopic screening, which was 67% (range, 40%-90%) according to a previous report (Table 1).^15^ The model assumed that false-positive results would result in loss of quality of life but could be corrected with an endoscopic reexamination at an additional cost. The sensitivity and specificity of endoscopic examination for EC were 96% (range, 88%-99%) and 90% (range, 59%-100%), respectively, according to previous studies in areas in China where risk of EC is high.^11,30,31^ The sensitivity and specificity of endoscopic examination for GC were assumed to be 89% (range, 70%-98%) and 100% (range, 90%-100%), respectively, based on the published literature from other countries where risk for GC is high and other economic evaluation studies of GC^8,12,32,33,34^ because of the lack of available data in China. Clinically significant complications (eg, bleeding or perforation) are rare among individuals undergoing diagnostic endoscopic examination.^42,43^ The complication rate in the model was estimated to be 0.009% (range, 0%-0.2%) based on the trial by Chen et al^23,24^ and other published studies (Table 2).^32,33,35^

**Table 1.** Estimates of Parameters Used in the Model

Parameter	Base-case value	Range	Distribution	Source
Compliance with screening	0.49	0.30-0.80	Triangular (0.30, 0.49, 0.80)	Wei et al,^18^ 2015; Feng et al,^28^ 2015; Wang et al,^29^ 2015
Compliance with reexamination	0.67	0.40-0.90	Triangular (0.40, 0.67, 0.90)	Yang et al,^15^ 2015
Endoscopic examination characteristics				
Sensitivity for EC	0.96	0.88-0.99	Triangular (0.88, 0.96, 0.99)	Chang et al,^11^ 2012; Dawsey et al,^30^ 1998; Nagami et al,^31^ 2014
Specificity for EC	0.90	0.59-1.00	Triangular (0.59, 0.90, 1.00)	Chang et al,^11^ 2012; Dawsey et al,^30^ 1998; Nagami et al,^31^ 2014
Sensitivity for GC	0.89	0.70-0.98	Triangular (0.70, 0.89, 0.98)	Zhou et al,^12^ 2013; Hamashima et al,^8^ 2018; Yeh et al,^32^ 2016; Lee et al,^33^ 2007; Hamashima et al,^34^ 2013
Specificity for GC	1.00	0.90-1.00	Triangular (0.90, 1.00, 1.00)	Zhou et al,^12^ 2013; Hamashima et al,^8^ 2018; Yeh et al,^32^ 2016; Lee et al,^33^ 2007; Hamashima et al,^34^ 2013
Endoscopic examination complications	0.00009	0-0.002	Triangular (0, 0.00009, 0.002)	Zeng et al,^24^ 2020; Yeh et al,^32^ 2016; Lee et al,^33^ 2007; Espino et al,^35^ 2012
Annual self-initiated examination				
Severe esophageal dysplasia and CIS or HGIN and CIS	0.01	0.005-0.02	Triangular (0.005, 0.01, 0.02)	Chang et al,^11^ 2012
Early EC or GC	0.20	0.10-0.40	Triangular (0.10, 0.20, 0.40)	Chang et al,^11^ 2012
Advanced EC or GC	0.70	0.56-0.90	Triangular (0.56, 0.70, 0.90)	Chang et al,^11^ 2012
Compliance with treatment				
Severe esophageal dysplasia or CIS	0.7458	0.5625-0.9654	β (31.72, 10.81)	Chen et al,^23^ 2017
Early EC	0.9405	0.7149-1.0000	β (8.17, 0.52)	Chen et al,^23^ 2017
Advanced EC	0.9643	0.8393-1.0000	β (16.99, 0.63)	Chen et al,^23^ 2017
HGIN or CIS	0.5455	0.4425-0.7746	β (92.43, 77.01)	Chen et al,^23^ 2017
Early GC	0.9000	0.6792-1.0000	β (12.41, 1.38)	Chen et al,^23^ 2017
Advanced GC	0.9643	0.8393-1.0000	β (16.99, 0.63)	Chen et al,^23^ 2017
Costs, $				
Screening mobilization and administration per capita	1.05	±50%	γ (0.16, 0.15)	Chen et al,^23^ 2017
Endoscopic examination	47.87	±50%	γ (46.57, 0.97)	Chen et al,^23^ 2017
Treatment for endoscopic complications	113.68	±50%	γ (5.82, 0.05)	Chen et al,^23^ 2017
Initial treatment				
Severe esophageal dysplasia or CIS	1604	±50%	γ (3.33, 0.002)	Yang et al,^36^ 2018
Early EC	7732	±50%	γ (2.33, 3.01)	Yang et al,^36^ 2018
Advanced EC	7320	±50%	γ (3.33, 4.55)	Yang et al,^36^ 2018
HGIN or CIS	1423	±50%	γ (1.41, 9.88)	Yang et al,^36^ 2018
Early GC	7548	±50%	γ (4.61, 6.11)	Yang et al,^36^ 2018
Advanced GC	7086	±50%	γ (5.96, 8.41)	Yang et al,^36^ 2018
Annual health care				
Severe esophageal dysplasia or CIS	216	±50%	γ (1.22, 0.006)	Yang et al,^36^ 2018
Early EC	367	±50%	γ (1.23, 0.003)	Yang et al,^36^ 2018
Advanced EC	342	±50%	γ (2.05, 0.006)	Yang et al,^36^ 2018
HGIN or CIS	243	±50%	γ (1.24, 0.005)	Yang et al,^36^ 2018
Early GC	409	±50%	γ (1.18, 0.003)	Yang et al,^36^ 2018
Advanced GC	435	±50%	γ (1.19, 0.003)	Yang et al,^36^ 2018
Utility scores				
Mild esophageal dysplasia	1.00	0.98-1.00	Triangular (0.98, 1.00, 1.00)	Sharaiha et al,^37^ 2014; Inadomi et al,^38^ 2009
Moderate esophageal dysplasia	1.00	0.98-1.00	Triangular (0.98, 1.00, 1.00)	Sharaiha et al,^37^ 2014; Inadomi et al,^38^ 2009
Severe esophageal dysplasia or CIS	0.84	0.79-0.89	β (3.57, 0.68)	Liu et al,^39^ 2018
Early EC	0.70	0.66-0.74	β (2.63, 1.13)	Liu et al,^39^ 2018
Advanced EC	0.61	0.56-0.66	β (1.12, 0.71)	Liu et al,^39^ 2018
LGIN	1.00	0.98-1.00	Triangular (0.98, 1.00, 1.00)	Sharaiha et al,^37^ 2014; Inadomi et al,^38^ 2009
HGIN or CIS	0.92	0.86-0.99	β (2.53, 0.22)	Xia et al,^40^ 2020
Early GC	0.75	0.71-0.78	β (3.15, 1.05)	Xia et al,^40^ 2020
Advanced GC	0.57	0.53-0.62	β (1.35, 1.02)	Xia et al,^40^ 2020
Discount rate	0.05	0-0.08	NA	Weinstein et al,^41^ 1996

Abbreviations: CIS, carcinoma in situ; EC, esophageal cancer; GC, gastric cancer; HGIN, high-grade intraepithelial neoplasia; LGIN, low-grade intraepithelial neoplasia; NA, not applicable.

**Table 2.** Base-Case Cost-effectiveness Results Compared Among Different Strategies by Initial Screening Age Among 100 000 Cohort Members^a^

Initial screening age, strategy	QALYs	Incremental QALYs		Cost ($, thousand)	Incremental cost ($, thousand)		ICER ($/QALY)	
		Vs no screening	Vs the next most effective strategy^b^		Vs no screening	Vs the next most effective strategy^b^	Vs no screening	Vs the next most effective strategy^b^
40-44 y								
No screening	1 659 260	NA	NA	25 035	NA	NA	NA	NA
Screening once per lifetime	1 660 347	1087	1087	28 334	3299	3299	3035	3035
Screening every 10 y	1 663 677	4417	3330	31 954	6919	3620	1566	1087
Screening every 5 y	1 666 345	7085	2668	36 707	11 672	4753	1647	1781
Screening every 3 y	1 668 371	9111	2026	42 218	17 183	5511	1886	2720
Screening every 2 y	1 669 622	10 362	1251	47 861	22 826	5643	2203	4511
45-49 y								
No screening	1 572 532	NA	NA	26 817	NA	NA	NA	NA
Screening once per lifetime	1 574 161	1629	1629	30 347	3530	3530	2167	2167
Screening every 10 y	1 576 941	4409	2780	33 961	7144	3614	1620	1300
Screening every 5 y	1 579 145	6613	2204	37 745	10 928	3784	1653	1717
Screening every 3 y	1 580 974	8442	1829	42 531	15 714	4786	1861	2617
Screening every 2 y	1 582 334	9802	1360	48 190	21 373	5659	2180	4161
50-54 y								
No screening	1 468 506	NA	NA	30 229	NA	NA	NA	NA
Screening once per lifetime	1 470 890	2384	2384	34 142	3913	3913	1641	1641
Screening every 10 y	1 472 583	4077	1693	36 373	6144	2231	1507	1318
Screening every 5 y	1 474 773	6267	2190	40 356	10 127	3983	1616	1819
Screening every 3 y	1 476 325	7819	1552	44 120	13 891	3764	1777	2425
Screening every 2 y	1 477 651	9145	1326	49 010	18 781	4890	2054	3688
55-59 y								
No screening	1 342 830	NA	NA	36 095	NA	NA	NA	NA
Screening once per lifetime	1 346 031	3201	3201	40 491	4396	4396	1373	1373
Screening every 10 y	1 347 264	4434	1233	42 811	6716	2320	1515	1882
Screening every 5 y	1 348 747	5917	1483	45 240	9145	2429	1546	1638
Screening every 3 y	1 350 365	7535	1618	49 171	13 076	3931	1735	2430
Screening every 2 y	1 351 491	8661	1126	52 843	16 748	3672	1934	3261
60-64 y								
No screening	1 195 742	NA	NA	45 876	NA	NA	NA	NA
Screening once per lifetime	1 199 618	3876	3876	51 081	5205	5205	1343	1343
Screening every 5 y	1 201 091	5349	1473	53 970	8094	2889	1513	1961
Screening every 3 y	1 202 293	6551	1202	56 498	10 622	2528	1621	2103
Screening every 2 y	1 203 210	7468	917	58 779	12 903	2281	1728	2487
65-69 y								
No screening	1 025 119	NA	NA	60 442	NA	NA	NA	NA
Screening once per lifetime	1 029 058	3939	3939	67 233	6791	6791	1724	1724
Screening every 2 y	1 030 594	5475	1536	70 810	10 368	3577	1894	2329

Abbreviations: ICER, incremental cost-effectiveness ratio; NA, not applicable; QALY, quality-adjusted life-year.

^a^ QALYs, costs, and ICERs are expressed as the values in 2019.

^b^ Compared with the next most effective strategy at the same initial screening age.

The prevalence rates of EC- and GC-related health states were estimated based on baseline screening reports from the trial by Chen et al^23^ in areas where the risk of these cancers is high (eTable 1 in the Supplement); these reports were used to determine initial distributions of cohort members across health states in the model. A wide range was set for each rate to cover the values reported in these areas by referring to previous studies from China.^29,44,45,46^ The annual transition probabilities were derived from published observational studies concerning the natural history of EC and GC and economic evaluation studies of EC and GC (eTable 2 in the Supplement).^15,47,48,49^

The model assumed that in the absence of active screening, individuals with severe esophageal dysplasia and carcinoma in situ, high-grade gastric intraepithelial neoplasia and carcinoma in situ, or EC or GC would receive a diagnosis on the basis of self-initiated examinations according to state-specific probabilities (Table 1).^11^ Individuals with these diagnoses would receive state-specific treatments, whereas individuals who did not receive these diagnoses would remain untreated. The rates of state-specific compliance with treatment were calculated for the proportion of screened patients who actually completed the entire treatment procedure in areas where the risk of UGIC is high in the trial by Chen et al^23^ (eTable 3 in the Supplement). The state-specific probabilities of recurrence after treatment and cancer-related mortality rates for advanced EC and GC were estimated based on the survival rates among patients with EC and GC (eTable 2 in the Supplement),^15^ whereas age-specific natural background death rates were obtained from the China Population & Employment Statistics Yearbook, 2019.^50^

Costs were converted from Chinese renminbi to 2019 US dollars (US $1 = ¥6.8968 in 2019). The cost of screening included screening mobilization and administration costs, endoscopic examination costs, and costs of treatment for endoscopic complications, all of which were obtained from the 7 study centers that participated in the trial by Chen et al^23^ (eTable 4 in the Supplement). The cost of EC- and GC-related treatment included the initial treatment cost after detection of the cancer and the subsequent annual health care cost after treatment, both of which were obtained from the survey included in the trial by Chen et al^23^ that was administered to assess the economic burden of UGIC in China.^36^ Calculation details are shown in eTable 5 in the Supplement.

The health outcome was utility-weighted life expectancy expressed as quality-adjusted life-years (QALYs). Utility scores of EC- and GC-related health states were obtained from the survey included in the trial by Chen et al^23^ that was administered to assess the quality of life of patients with UGIC in China (eTable 6 in the Supplement).^39,40^ Considering that patients diagnosed with mild esophageal dysplasia, moderate esophageal dysplasia, and low-grade gastric intraepithelial neoplasia do not have symptoms, we used a utility score of 1 in the base-case analysis and set a range of 0.98 to 1 in the sensitivity analyses.^37,38^ We assumed a discount rate of 5% (range, 0%-8%) for both QALYs and costs.^41^ The choice of distribution for all parameters was based on consideration of the properties of the parameters and the data informing the parameters.

### Statistical Analysis

#### Effectiveness and Cost-effectiveness Evaluation

Expected QALYs and costs of each strategy were obtained from the model. We evaluated the cost-effectiveness of screening strategies at different initial screening ages with 2 approaches. First, we calculated the incremental cost-effectiveness ratio (ICER), defined as incremental costs per QALY gained of each screening strategy compared with no screening. Second, we calculated the ICER of each screening strategy compared with the next most effective screening strategy to identify the optimal strategy at each initial screening age. Because of the lack of data regarding the willingness to pay in the population of China, we adopted the cost-effectiveness definition used by the World Health Organization. Highly cost-effective was defined as an ICER less than 1 time the per capita gross domestic product (GDP) in China; cost-effective, an ICER of 1 to 3 times the per capita GDP; and not cost-effective, and an ICER greater than 3 times the per capita GDP.^51^ The per capita GDP in China in 2019 was US $10 276.

#### Uncertainty and Sensitivity Analyses

Univariate sensitivity analyses of all parameters within their respective ranges were performed to identify the main sensitivity parameters. Probabilistic sensitivity analyses were further conducted to determine the probability of each screening strategy being cost-effective compared with no screening and the probability of each strategy being optimal compared with all other strategies.

## Results

### Base-Case Results

The study included a hypothetical sample of 100 000 individuals aged 40 to 69 years. All 5 screening strategies increased QALYs and costs compared with no screening at the same initial screening age (Table 2) by 1087 to 10 362 QALYs and US $3 299 000 to $22 826 000 for a cohort of 100 000 participants over a lifetime, and the corresponding ICERs were US $1343 to $3035 per QALY, which were lower than the per capita GDP ($10 276). Therefore, all screening strategies would be more cost-effective than no screening.

Further comparisons of the screening strategies were performed, and more frequent screening would be associated with a gain of more QALYs but with higher costs (Table 2). When a screening strategy was compared with the next most effective screening strategy in the cohort with the same initial screening age, 917 to 3939 QALYs would be gained and an additional cost of US $2 231 000 to $6 791 000 gained for a cohort of 100 000 participants over a lifetime; the corresponding ICERs were US $1087 to $4511 per QALY, which was lower than the per capita GDP. Therefore, the high-frequency screening strategy would be more cost-effective than the low-frequency screening strategy, and screening every 2 years would be the optimal strategy at each initial screening age. Screening every 2 years and screening once per lifetime had the most incremental QALYs at the initial screening ages of 40 to 44 years and 65 to 69 years, respectively.

### Sensitivity Analysis Results

Univariate sensitivity analyses revealed that the results remained largely unchanged over the plausible range of each parameter. The ICER upper limits of the most sensitive parameters of each screening strategy at different initial screening ages are shown in Table 3. When a screening strategy was compared with no screening, only varying utility scores of EC- and GC-related health states resulted in ICERs exceeding the per capita GDP at initial screening ages of 40 to 44 years and 65 to 69 years. When a screening strategy was compared with the next most effective screening strategy, varying utility scores of EC- and GC-related health states resulted in ICERs at some initial screening ages exceeding 3 times the per capita GDP, and varying compliance with screening resulted in ICERs of screening every 2 years vs screening every 3 years exceeding the per capita GDP. Univariate sensitivity analyses demonstrated the stability of the cost-effectiveness and ranking of the cost-effectiveness of the screening strategies.

**Table 3.** Upper Limits of Incremental Cost-effectiveness Ratio for Each Screening Strategy in Univariate Sensitivity Analyses by Initial Screening Age^a^

Initial screening age and screening strategy	Utility scores^b^		Compliance with screening		Discount rate		Prevalences of EC- and GC-related health states^b^		Costs of screening^c^		Annual transition probability from severe esophageal dysplasia or CIS to early EC	
	Vs no screening	Vs the next most effective strategy	Vs no screening	Vs the next most effective strategy	Vs no screening	Vs the next most effective strategy	Vs no screening	Vs the next most effective strategy	Vs no screening	Vs the next most effective strategy	Vs no screening	Vs the next most effective strategy
40-44 y												
Screening once per lifetime	11 778^d^	11 778^d^	4016	4016	6671	6671	4711	4711	4422	4422	4592	4592
Screening every 10 y	4498	2877	1883	1271	3440	2144	1737	1129	2272	1570	2186	1658
Screening every 5 y	5116	6395	1845	2277	3467	3513	1811	1927	2397	2603	2308	2513
Screening every 3 y	6202	11 273^d^	2085	5204	3871	5260	2082	3003	2758	4022	2609	3659
Screening every 2 y	7452	19 296^d^	2619	13 156^d^	4480	8663	2451	5127	3240	6747	2996	5740
45-49												
Screening once per lifetime	6153	6153	2760	2760	4486	4486	3448	3448	3092	3092	2677	2677
Screening every 10 y	5236	4571	1912	1473	3248	2358	1934	1389	2304	1842	2259	1986
Screening every 5 y	5310	5455	1853	2155	3246	3241	1949	1979	2370	2502	2284	2333
Screening every 3 y	6217	10 190	2020	4874	3583	4782	2206	3090	2687	3830	2539	3455
Screening every 2 y	7678	22 118^d^	2552	10 504^d^	4128	7457	2605	5002	3163	6125	2936	5387
50-54 y												
Screening once per lifetime	3976	3976	1674	1974	3216	3216	2609	2609	2296	2296	2134	2134
Screening every 10 y	4253	4844	1564	1409	2897	2367	2010	1479	2122	1876	2069	1972
Screening every 5 y	5210	7982	1850	2752	2998	3197	2091	2219	2292	2609	2238	2560
Screening every 3 y	5803	8366	2233	6715	3252	4255	2320	3550	2542	3197	2424	3161
Screening every 2 y	7025	17 484^d^	2821	12 807^d^	3695	6278	2710	4887	2956	5401	2768	4787
55-59 y												
Screening once per lifetime	3271	3271	1394	1394	2601	2601	2127	2127	1882	1882	1920	1920
Screening every 10 y	4841	52 896^e^	1527	2043	2746	3194	2151	2191	2084	2608	2194	2954
Screening every 5 y	5026	5620	1680	2573	2777	2868	2186	2277	2155	2366	2210	2258
Screening every 3 y	6162	13 002^d^	2076	5658	3046	4045	2476	3421	2443	3496	2455	3346
Screening every 2 y	6920	12 320^d^	2523	13 669^d^	3357	5377	2814	5008	2745	4770	2694	4255
60-64 y												
Screening once per lifetime	3635	3635	1360	1360	2476	2476	1995	1995	1794	1794	2054	2054
Screening every 5 y	5310	31 253^e^	1566	2611	2697	3324	2235	2736	2055	2741	2324	3033
Screening every 3 y	6082	11 374^d^	1805	4871	2847	3515	2448	3321	2228	3001	2459	3044
Screening every 2 y	6492	9464	2076	11 769^d^	3011	4159	2683	4334	2399	3621	2586	3459
65-69 y												
Screening once per lifetime	8653	8653	1741	1741	3128	3128	2375	2375	2185	2185	3053	3053
Screening every 2 y	14 302^d^	25 436^d^	2019	4801	3372	4011	2747	3644	2456	3153	3313	3959

Abbreviations: CIS, carcinoma in situ; EC, esophageal cancer; GC, gastric cancer; ICER, incremental cost-effectiveness ratio.

^a^ Only main sensitive parameters are shown; ICERs are expressed as the value in 2019.

^b^ As a set of parameters, all the parameters changed simultaneously with a positive correlation in univariate sensitivity analyses.

^c^ Including screening mobilization and administration costs, endoscopic examination costs, and treatment costs for endoscopic complications. The 3 parameters changed simultaneously with a positive correlation in univariate sensitivity analyses.

^d^ The upper limit of ICER was higher than the per capita GDP but lower than 3 times the per capita GDP.

^e^ The upper limit of ICER was higher than 3 times the per capita GDP.

**Figure 1.**
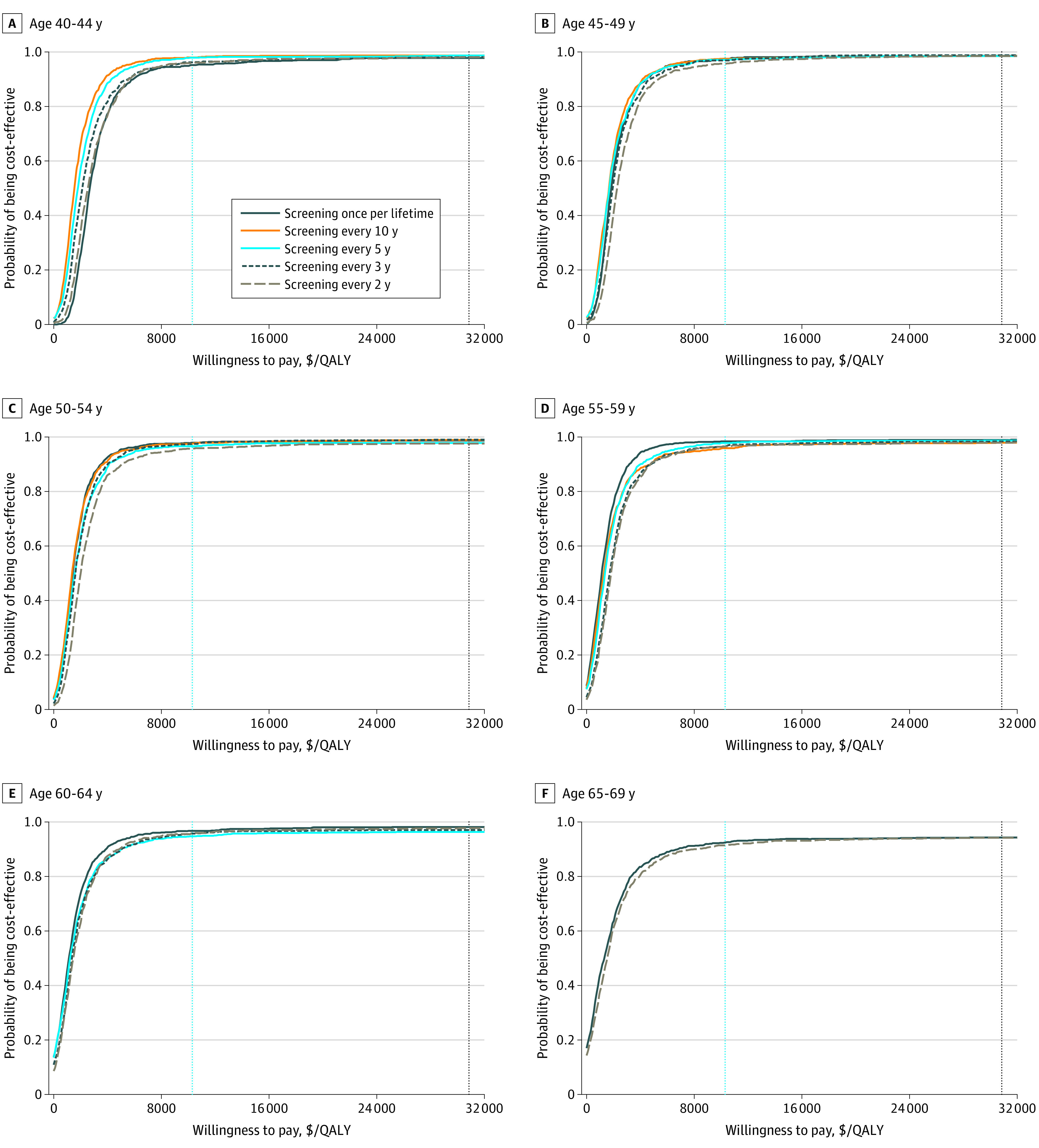
Cost-effectiveness Acceptability Curves of All Screening Strategies Compared With No Screening by Initial Screening Age Dashed vertical blue lines represent per capita gross domestic product (GDP); dashed vertical black lines represent 3 times per capita GDP. QALY indicates quality-adjusted life-year.

**Figure 2.**
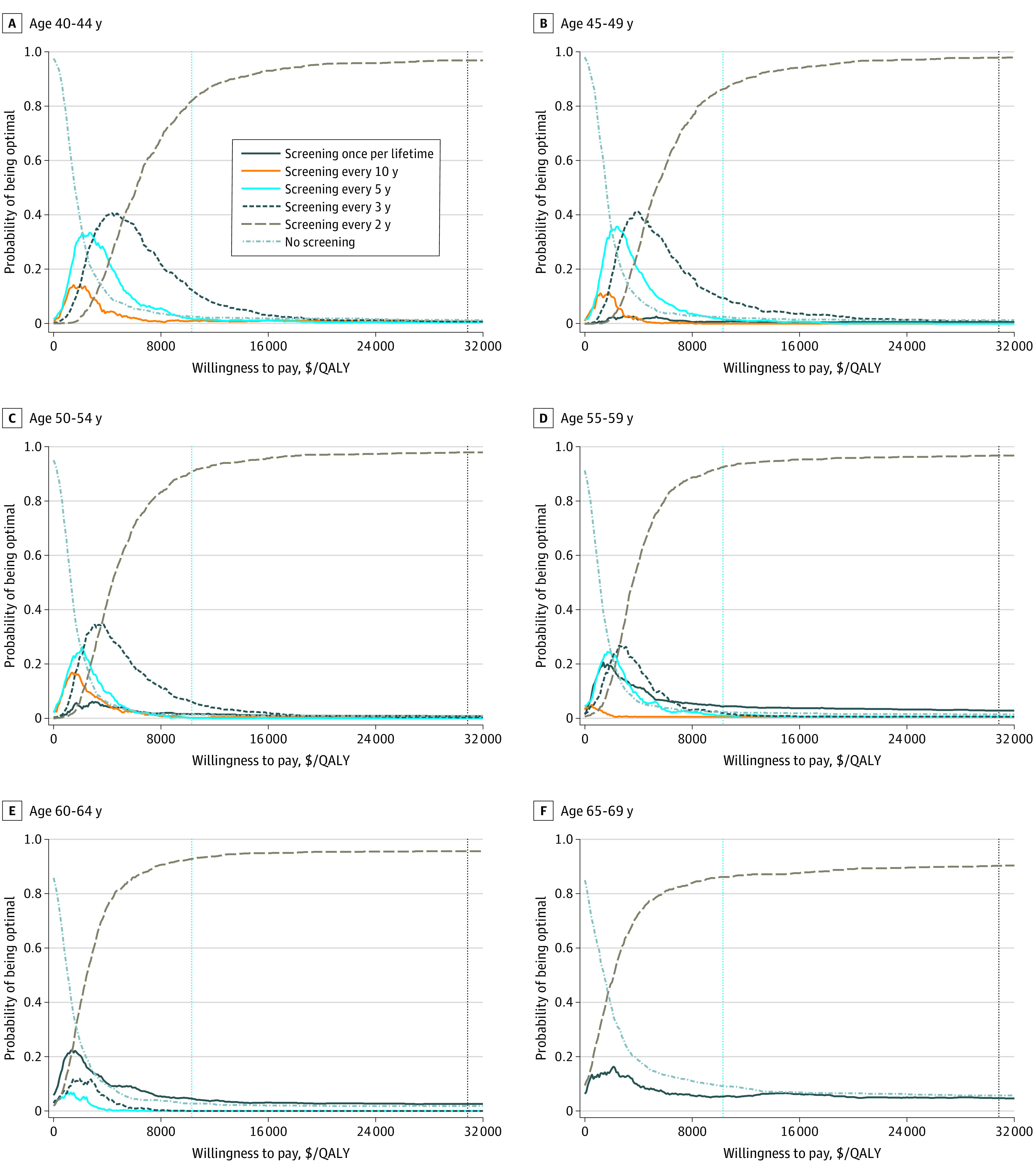
Cost-effectiveness Acceptability Curves of All Strategies Competing With Each Other by Initial Screening Age Dashed vertical blue lines represent per capita gross domestic product (GDP); dashed vertical black lines represent 3 times per capita GDP. QALY indicates quality-adjusted life-year.

The results of the probabilistic sensitivity analyses of each screening strategy compared with no screening are shown in Figure 1. The probability of each screening strategy being cost-effective increased as the willingness-to-pay threshold increased and reached 91% to 98% even at a willingness-to-pay threshold of the per capita GDP. The results of the probabilistic sensitivity analyses conducted for each strategy compared with all other strategies are shown in Figure 2. Screening every 2 years maintained its dominance from a willingness-to-pay threshold of less than the per capita GDP to a willingness-to-pay threshold equal to 3 times the per capita GDP at each initial screening age, with probabilities of 82% to 93% and 90% to 98% for being optimal at 1 and 3 times per capita GDP, respectively. Therefore, all screening strategies would be more cost-effective than no screening, and screening every 2 years would be the optimal strategy at a willingness-to-pay threshold of 3 times the per capita GDP.

## Discussion

Upper gastrointestinal tract cancer continues to be a major public health burden in areas in China where incidence and mortality rate for this cancer are higher than those in other areas. The present study targeted local residents aged 40 to 69 years in areas where the risk of UGIC is high who were most likely to benefit from endoscopic screening according to the age characteristics of UGIC incidence and life expectancy in China, and this population accounts for a large proportion of patients with UGIC in China. Our base-case results suggest that combined endoscopic screening for EC and GC would be more cost-effective than no screening regardless of the initial screening age or screening frequency; this finding is consistent with the conclusion reported in a systematic review.^52^ Screening every 2 years would be the optimal strategy, and an initial screening age of 40 to 44 years was associated with the most health benefits. A screening frequency of 1 to 5 years has been previously proposed,^53^ with the optimal interval being less than 3 years.^54^ Most cases of early UGIC that were missed at the first endoscopic examination and subsequently identified in the second screening within 3 years were still amenable to curative surgery,^55^ which could theoretically increase the sensitivity of endoscopic examination after 2 consecutive screenings.^13^ The optimal strategy in this study was similar to the guidelines recommended in Japan and South Korea,^8,9^ where the incidence rates are as high as those in China. Areas in China where the risk of UGIC is high are usually rural and have limited health resources and an underdeveloped economy.^56,57^ Policy makers should consider the cost and effectiveness of the screening strategy, local economic level, and disease burden of UGIC when choosing appropriate screening strategies. If screening once per lifetime, which was the least expensive screening strategy, is preferable, the optimal initial screening age suggested by this study is 65 to 69 years.

Univariate sensitivity analyses revealed that utility scores of EC- and GC-related health states and compliance with screening were the 2 main sensitivity parameters. In this study, because most patients diagnosed with mild esophageal dysplasia, moderate esophageal dysplasia, or low-grade gastric intraepithelial neoplasia did not have symptoms, we used a utility score of 1 in the base-case analysis and a range of 0.98 to 1 in the sensitivity analyses according to other reports.^37,38^ Whether any decrements in the quality of life could be present in patients diagnosed with mild esophageal dysplasia, moderate esophageal dysplasia, or low-grade intraepithelial neoplasia if they were asymptomatic and received education regarding their true cancer risk is unknown.^17^ Although previous studies have found that patients with precancerous lesions have a poorer quality of life than the general population,^58,59^ whether the decrement in the quality of life is caused by coexisting symptoms (eg, esophagitis, gastric ulcers) or the patient’s perception of cancer risk remains unknown.^60^ Future studies should focus on the effect on the quality of life among those diagnosed with precancerous lesions. Compliance with screening affected whether the optimal strategy was screening every 2 years or screening every 3 years at a willingness-to-pay threshold of the per capita GDP. Endoscopic screening may be associated with reduced incidence and mortality of UGIC, and improving compliance among the target population is critical for achieving prevention effectiveness. The compliance with screening in this study was only 49%, which was derived from the areas with the highest incidence of UGIC in China^18^; these areas have the most longstanding promotion times and the most abundant experience with endoscopic screening and have residents with the highest cognitive understanding of endoscopic screening. Endoscopic screening is relatively expensive and slightly invasive, resulting in discomfort when individuals are examined. In addition, most of the target population, aged 40 to 69 years (especially men), in areas where there is high risk of UGIC are the primary sources of family income and migrate elsewhere for work. Therefore, compliance with endoscopic screening for UGIC is still low in China, and further popularization and promotion of endoscopic screening are needed to improve compliance.

### Limitations

This study has limitations. The accuracy of the model depends on the accuracy of parameter estimates. First, base-case initial probabilities of EC- and GC-related health states were derived from the study by Chen et al.^23^ Residents of areas where there is high risk of UGIC enrolled in the project may have had a higher incidence of this cancer; thus, the estimated parameters may not represent the status of the entire region. Second, annual progression or regression transition probabilities of health states should increase or decrease with age in the real world. However, those between specific precancerous lesion states were fixed owing to the lack of relevant observational data. As a result, the cost-effectiveness of screening strategies may be overestimated in younger age groups and underestimated in older age groups. Third, compliance with different screening frequencies was assumed to be consistent in the model. In the real world, a higher screening frequency may be associated with a reduction in compliance because of patients’ concerns about pain and sedation and competing life demands.^61,62^ Fourth, annual screening was not considered as an alternative strategy in this study because it has not been recommended in any country until now. In addition, the burden may be difficult to address in countries with low GDP.

## Conclusions

To our knowledge, this is the first comprehensive cost-effectiveness analysis of endoscopic screening for both EC and GC in China. The findings from the present study suggest that from the perspective of the health care system, combined endoscopic screening for EC and GC would be highly cost-effective for people aged 40 to 69 years in areas of China where there is a high risk of UGIC; screening every 2 years would be the optimal strategy. The findings provide important evidence for policies targeting the prevention and control of UGIC in China.

## Notes

Supplement.
eMethods.
**eFigure 1.** Flowchart of the Overall Participants in the Multicenter Randomized Trial Project **eFigure 2.** Flowchart of the Participants in the High-Risk Areas **eFigure 3.** Flowchart of the Screening and Reexamination **eFigure 4.** Markov Model of Upper Gastrointestinal Tract Cancer (Including Esophageal Cancer and Gastric Cancer) Progression **eFigure 5.** Model Projected Age-Specific GC Incidence and Mortality Rates Compared to 2 Folds of Observed National GC Incidence and Mortality Rates in 2015 **eFigure 6.** Model Projected Age-Specific EC Incidence and Mortality Rates Compared to 2 Folds of Observed National EC Incidence and Mortality Rates in 2015 **eTable 1.** Prevalence Rates (%) of EC/GC-Related Health States Used in the Model, by Initial Screening Age **eTable 2.** Annual Transition Probabilities Used in the Model **eTable 3.** State-Specific Compliances with Treatment Estimated Based on Our Project **eTable 4.** Costs of Screening Estimated Based on Our Project **eTable 5.** Costs of EC/GC-Related Treatment in Different Disease Progression Stages Based on the Survey Included in Our Project (US$) **eTable 6.** Utility Scores of EC/GC-Related Health States Based on the Survey Included in Our Project
eReferences.

## References

[1] Sung H, Ferlay J, Siegel RL, . Global Cancer Statistics 2020: GLOBOCAN estimates of incidence and mortality worldwide for 36 cancers in 185 countries. CA Cancer J Clin. 2021;71(3):209-249. doi:10.3322/caac.21660 33538338

[2] Wei WQ, Yang J, Zhang SW, Chen WQ, Qiao YL. Esophageal cancer mortality trends during the last 30 years in high-risk areas in China: comparison of results from national death surveys conducted in the 1970’s, 1990’s and 2004-2005. Asian Pac J Cancer Prev. 2011;12(7):1821-1826. 22126573

[3] Lin Y, Totsuka Y, Shan B, . Esophageal cancer in high-risk areas of China: research progress and challenges. Ann Epidemiol. 2017;27(3):215-221. doi:10.1016/j.annepidem.2016.11.004 28007352

[4] Li JY, Liu BQ, Li GY, Chen ZJ, Sun XI, Rong SD. Atlas of cancer mortality in the People’s Republic of China: an aid for cancer control and research. Int J Epidemiol. 1981;10(2):127-133. doi:10.1093/ije/10.2.127 7287273

[5] Zeng H, Chen W, Zheng R, . Changing cancer survival in China during 2003-15: a pooled analysis of 17 population-based cancer registries. Lancet Glob Health. 2018;6(5):e555-e567. doi:10.1016/S2214-109X(18)30127-X 29653628

[6] Wang GQ, Jiao GG, Chang FB, . Long-term results of operation for 420 patients with early squamous cell esophageal carcinoma discovered by screening. Ann Thorac Surg. 2004;77(5):1740-1744. doi:10.1016/j.athoracsur.2003.10.098 15111177

[7] Katai H, Ishikawa T, Akazawa K, ; Registration Committee of the Japanese Gastric Cancer Association. Five-year survival analysis of surgically resected gastric cancer cases in Japan: a retrospective analysis of more than 100,000 patients from the nationwide registry of the Japanese Gastric Cancer Association (2001-2007). Gastric Cancer. 2018;21(1):144-154. doi:10.1007/s10120-017-0716-7 28417260

[8] Hamashima C; Systematic Review Group and Guideline Development Group for Gastric Cancer Screening Guidelines. Update version of the Japanese guidelines for gastric cancer screening. Jpn J Clin Oncol. 2018;48(7):673-683. doi:10.1093/jjco/hyy077 29889263

[9] Kim Y, Jun JK, Choi KS, Lee HY, Park EC. Overview of the National Cancer screening programme and the cancer screening status in Korea. Asian Pac J Cancer Prev. 2011;12(3):725-730. doi:10.1097/01.cad.0000390767.85658.83 21627372

[10] Hirota WK, Zuckerman MJ, Adler DG, ; Standards of Practice Committee, American Society for Gastrointestinal Endoscopy. ASGE guideline: the role of endoscopy in the surveillance of premalignant conditions of the upper GI tract. Gastrointest Endosc. 2006;63(4):570-580. doi:10.1016/j.gie.2006.02.004 16564854

[11] Chang HS, Park EC, Chung W, . Comparing endoscopy and upper gastrointestinal x-ray for gastric cancer screening in South Korea: a cost-utility analysis. Asian Pac J Cancer Prev. 2012;13(6):2721-2728. doi:10.7314/APJCP.2012.13.6.2721 22938448

[12] Zhou HJ, Dan YY, Naidoo N, Li SC, Yeoh KG. A cost-effectiveness analysis evaluating endoscopic surveillance for gastric cancer for populations with low to intermediate risk. PLoS One. 2013;8(12):e83959. doi:10.1371/journal.pone.0083959 24386314 PMC3873968

[13] Areia M, Spaander MC, Kuipers EJ, Dinis-Ribeiro M. Endoscopic screening for gastric cancer: a cost-utility analysis for countries with an intermediate gastric cancer risk. United European Gastroenterol J. 2018;6(2):192-202. doi:10.1177/2050640617722902 29511549 PMC5833230

[14] Saumoy M, Schneider Y, Shen N, Kahaleh M, Sharaiha RZ, Shah SC. Cost effectiveness of gastric cancer screening according to race and ethnicity. Gastroenterology. 2018;155(3):648-660. doi:10.1053/j.gastro.2018.05.026 29778607

[15] Yang J, Wei WQ, Niu J, Liu ZC, Yang CX, Qiao YL. Cost-benefit analysis of esophageal cancer endoscopic screening in high-risk areas of China. World J Gastroenterol. 2012;18(20):2493-2501. doi:10.3748/wjg.v18.i20.2493 22654446 PMC3360447

[16] Hur C, Choi SE, Kong CY, . High-resolution microendoscopy for esophageal cancer screening in China: a cost-effectiveness analysis. World J Gastroenterol. 2015;21(18):5513-5523. doi:10.3748/wjg.v21.i18.5513 25987774 PMC4427673

[17] Gupta N, Bansal A, Wani SB, Gaddam S, Rastogi A, Sharma P. Endoscopy for upper GI cancer screening in the general population: a cost-utility analysis. Gastrointest Endosc. 2011;74(3):610-624.e2. doi:10.1016/j.gie.2011.05.001 21741639

[18] Wei WQ, Chen ZF, He YT, . Long-term follow-up of a community assignment, one-time endoscopic screening study of esophageal cancer in China. J Clin Oncol. 2015;33(17):1951-1957. doi:10.1200/JCO.2014.58.0423 25940715 PMC4881309

[19] Chen R, Liu Y, Song G, . Effectiveness of one-time endoscopic screening programme in prevention of upper gastrointestinal cancer in China: a multicentre population-based cohort study. Gut. 2021;70(2):251-260. doi:10.1136/gutjnl-2019-320200 32241902 PMC7815635

[20] Zhang N, Li Y, Chang X, . Long-term effectiveness of one-time endoscopic screening for esophageal cancer: a community-based study in rural China. Cancer. 2020;126(20):4511-4520. doi:10.1002/cncr.33119 33460056

[21] Liu M, He Z, Guo C, . Effectiveness of intensive endoscopic screening for esophageal cancer in China: a community-based study. Am J Epidemiol. 2019;188(4):776-784. doi:10.1093/aje/kwy291 30608546

[22] Zhang X, Li M, Chen S, . Endoscopic screening in Asian countries is associated with reduced gastric cancer mortality: a meta-analysis and systematic review. Gastroenterology. 2018;155(2):347-354.e9. doi:10.1053/j.gastro.2018.04.026 29723507

[23] Chen W, Zeng H, Chen R, . Evaluating efficacy of screening for upper gastrointestinal cancer in China: a study protocol for a randomized controlled trial. Chin J Cancer Res. 2017;29(4):294-302. doi:10.21147/j.issn.1000-9604.2017.04.02 28947861 PMC5592817

[24] Zeng H, Sun K, Cao M, . Initial results from a multi-center population-based cluster randomized trial of esophageal and gastric cancer screening in China. BMC Gastroenterol. 2020;20(1):398. doi:10.1186/s12876-020-01517-3 33228549 PMC7686770

[25] Husereau D, Drummond M, Petrou S, ; ISPOR Health Economic Evaluation Publication Guidelines-CHEERS Good Reporting Practices Task Force. Consolidated Health Economic Evaluation Reporting Standards (CHEERS)—explanation and elaboration: a report of the ISPOR Health Economic Evaluation Publication Guidelines Good Reporting Practices Task Force. Value Health. 2013;16(2):231-250. doi:10.1016/j.jval.2013.02.002 23538175

[26] Chinese Medical Association, Chinese Society of Digestive Endoscopy, Chinese Anti Cancer Association, The Society of Tumor Endoscopy. Chinese expert consensus on screening and endoscopic management of early esophageal cancer (Beijing, 2014). Clin J Gastroenterol. 2015;20(4):220-240. doi:10.3969/j.issn.1008-7125.2015.04.006

[27] Chinese Medical Association, Chinese Society of Digestive Endoscopy, Chinese Anti-Cancer Association, The Society of Tumor Endoscopy. Chinese consensus on screening and endoscopic diagnosis and management of early gastric cancer (Changsha, 2014). Article in Chinese. Clin J Gastroenterol. 2014;19(7):408-427. doi:10.3969/j.issn.1008-7125.2014.07.006

[28] Feng H, Song G, Yang J, . Cost-effectiveness analysis of esophageal cancer once-in-a-lifetime endoscopic screening in high-risk areas of rural China. Article in Chinese. Zhonghua Zhong Liu Za Zhi. 2015;37(6):476-480. doi:10.3760/cma.j.issn.0253-3766.2015.06.017 26463155

[29] Wang M, Hao C, Zhao D, . Distribution of esophageal squamous cell cancer and precursor lesions in high-risk areas, Linzhou in Henan province and Feicheng in Shandong province of China, 2005-2009. Article in Chinese. Zhonghua Yu Fang Yi Xue Za Zhi. 2015;49(8):677-682. 26733024

[30] Dawsey SM, Fleischer DE, Wang GQ, . Mucosal iodine staining improves endoscopic visualization of squamous dysplasia and squamous cell carcinoma of the esophagus in Linxian, China. Cancer. 1998;83(2):220-231. doi:10.1002/(SICI)1097-0142(19980715)83:2<220::AID-CNCR4>3.0.CO;2-U 9669803

[31] Nagami Y, Tominaga K, Machida H, . Usefulness of non-magnifying narrow-band imaging in screening of early esophageal squamous cell carcinoma: a prospective comparative study using propensity score matching. Am J Gastroenterol. 2014;109(6):845-854. doi:10.1038/ajg.2014.94 24751580 PMC4050526

[32] Yeh JM, Hur C, Ward Z, Schrag D, Goldie SJ. Gastric adenocarcinoma screening and prevention in the era of new biomarker and endoscopic technologies: a cost-effectiveness analysis. Gut. 2016;65(4):563-574. doi:10.1136/gutjnl-2014-308588 25779597 PMC4573370

[33] Lee YC, Lin JT, Wu HM, . Cost-effectiveness analysis between primary and secondary preventive strategies for gastric cancer. Cancer Epidemiol Biomarkers Prev. 2007;16(5):875-885. doi:10.1158/1055-9965.EPI-06-0758 17507609

[34] Hamashima C, Okamoto M, Shabana M, Osaki Y, Kishimoto T. Sensitivity of endoscopic screening for gastric cancer by the incidence method. Int J Cancer. 2013;133(3):653-659. doi:10.1002/ijc.28065 23364866

[35] Espino A, Garcia X, Mac-Namara M, . 805 complications of gastrointestinal endoscopy in 85,391 procedures. Gastrointest Endosc. 2012;75(4)(suppl S):AB170. doi:10.1016/j.gie.2012.04.140

[36] Yang Z, Zeng H, Xia R, . Annual cost of illness of stomach and esophageal cancer patients in urban and rural areas in China: A multi-center study. Chin J Cancer Res. 2018;30(4):439-448. doi:10.21147/j.issn.1000-9604.2018.04.07 30210224 PMC6129568

[37] Sharaiha RZ, Freedberg DE, Abrams JA, Wang YC. Cost-effectiveness of chemoprevention with proton pump inhibitors in Barrett’s esophagus. Dig Dis Sci. 2014;59(6):1222-1230. doi:10.1007/s10620-014-3186-3 24795040 PMC4315516

[38] Inadomi JM, Somsouk M, Madanick RD, Thomas JP, Shaheen NJ. A cost-utility analysis of ablative therapy for Barrett’s esophagus. Gastroenterology. 2009;136(7):2101-2114.e1-e6. doi:10.1053/j.gastro.2009.02.062 19272389 PMC2693449

[39] Liu Q, Zeng H, Xia R, . Health-related quality of life of esophageal cancer patients in daily life after treatment: a multicenter cross-sectional study in China. Cancer Med. 2018;7(11):5803-5811. doi:10.1002/cam4.1817 30350456 PMC6247038

[40] Xia R, Zeng H, Liu Q, . Health-related quality of life and health utility score of patients with gastric cancer: a multi-centre cross-sectional survey in China. Eur J Cancer Care (Engl). 2020;29(6):e13283. doi:10.1111/ecc.13283 32602238

[41] Weinstein MC, Siegel JE, Gold MR, Kamlet MS, Russell LB. Recommendations of the Panel on Cost-effectiveness in Health and Medicine. JAMA. 1996;276(15):1253-1258. doi:10.1001/jama.1996.03540150055031 8849754

[42] Sieg A, Hachmoeller-Eisenbach U, Eisenbach T. Prospective evaluation of complications in outpatient GI endoscopy: a survey among German gastroenterologists. Gastrointest Endosc. 2001;53(6):620-627. doi:10.1067/mge.2001.114422 11323588

[43] Levy I, Gralnek IM. Complications of diagnostic colonoscopy, upper endoscopy, and enteroscopy. Best Pract Res Clin Gastroenterol. 2016;30(5):705-718. doi:10.1016/j.bpg.2016.09.005 27931631

[44] Zheng X, Mao X, Xu K, . Massive endoscopic screening for esophageal and gastric cancers in a high-risk area of China. PLoS One. 2015;10(12):e0145097. doi:10.1371/journal.pone.0145097 26699332 PMC4689398

[45] Liang S, Li K, Gong J, Wang J, Ma H, Wang G. Results of the endoscopic screening program of esophageal and gastric cardia cancers using iodine staining in Feicheng, Shandong Province, from 2006 to 2012. Article in Chinese. Zhonghua Zhong Liu Za Zhi. 2015;37(7):549-553. 26463335

[46] You WC. Intervention on gastric cancer and precancerous lesions—the practice of gastric cancer at high-risk area for 23 years. Journal of Peking University *(Health Sciences)*. 2006;38:565-570.

[47] Wang GQ, Abnet CC, Shen Q, . Histological precursors of oesophageal squamous cell carcinoma: results from a 13 year prospective follow up study in a high risk population. Gut. 2005;54(2):187-192. doi:10.1136/gut.2004.046631 15647178 PMC1774842

[48] Zhang Y, Zhang L, Pan KF, Prognosis of intestinal metaplasia and expressions of biomarkers in high-risk populations of gastric cancer in Shangdong province. Article in Chinese. World Chinese Journal of Digestology. 2006;14:2306-2310. doi:10.11569/wcjd.v14.i23.2306

[49] Wang LD, Yang HH, Fan ZM, . Cytological screening and 15 years’ follow-up (1986-2001) for early esophageal squamous cell carcinoma and precancerous lesions in a high-risk population in Anyang County, Henan Province, Northern China. Cancer Detect Prev. 2005;29(4):317-322. doi:10.1016/j.cdp.2005.06.004 16118042

[50] Department of Population and Employment Statistics, National Bureau of Statistics of China. China Population and Employment Statistics Yearbook. China Statistics Press; 2019.

[51] Walker DG, Hutubessy R, Beutels P. WHO guide for standardisation of economic evaluations of immunization programmes. Vaccine. 2010;28(11):2356-2359. doi:10.1016/j.vaccine.2009.06.035 19567247

[52] Areia M, Carvalho R, Cadime AT, Rocha Gonçalves F, Dinis-Ribeiro M. Screening for gastric cancer and surveillance of premalignant lesions: a systematic review of cost-effectiveness studies. Helicobacter. 2013;18(5):325-337. doi:10.1111/hel.12050 23566268

[53] Fukao A, Tsubono Y, Tsuji I, HIsamichi S, Sugahara N, Takano A. The evaluation of screening for gastric cancer in Miyagi Prefecture, Japan: a population-based case-control study. Int J Cancer. 1995;60(1):45-48. doi:10.1002/ijc.2910600106 7814150

[54] Kim YS, Park HA, Kim BS, Yook JH, Lee MS. Efficacy of screening for gastric cancer in a Korean adult population: a case-control study. J Korean Med Sci. 2000;15(5):510-515. doi:10.3346/jkms.2000.15.5.510 11068986 PMC3054678

[55] Hosokawa O, Tsuda S, Kidani E, . Diagnosis of gastric cancer up to three years after negative upper gastrointestinal endoscopy. Endoscopy. 1998;30(8):669-674. doi:10.1055/s-2007-1001386 9865554

[56] He YT, Hou J, Chen ZF, . Trends in incidence of esophageal and gastric cardia cancer in high-risk areas in China. Eur J Cancer Prev. 2008;17(2):71-76. doi:10.1097/CEJ.0b013e3282b6fd97 18287862

[57] Wang JM, Xu B, Hsieh CC, Jiang QW. Longitudinal trends of stomach cancer and esophageal cancer in Yangzhong County: a high-incidence rural area of China. Eur J Gastroenterol Hepatol. 2005;17(12):1339-1344. doi:10.1097/00042737-200512000-00012 16292087

[58] Crockett SD, Lippmann QK, Dellon ES, Shaheen NJ. Health-related quality of life in patients with Barrett’s esophagus: a systematic review. Clin Gastroenterol Hepatol. 2009;7(6):613-623. doi:10.1016/j.cgh.2009.02.024 19281858 PMC2693470

[59] Wang L, Shi JF, Zhu J, ; Health Economic Evaluation Working Group of the Cancer Screening Program in Urban China. Health-related quality of life and utility scores of patients with breast neoplasms in China: a multicenter cross-sectional survey. Breast. 2018;39:53-62. doi:10.1016/j.breast.2018.03.004 29597131

[60] Kruijshaar ME, Siersema PD, Janssens ACJW, Kerkhof M, Steyerberg EW, Essink-Bot ML; CYBAR Study Group. Patients with Barrett’s esophagus perceive their risk of developing esophageal adenocarcinoma as low. Gastrointest Endosc. 2007;65(1):26-30. doi:10.1016/j.gie.2006.05.030 17185076

[61] Oikonomidou E, Anastasiou F, Pilpilidis I, Kouroumalis E, Lionis C; Greek General Practice Dyspepsia Group. Upper gastrointestinal endoscopy for dyspepsia: exploratory study of factors influencing patient compliance in Greece. BMC Gastroenterol. 2011;11:11. doi:10.1186/1471-230X-11-11 21320314 PMC3042973

[62] Cao M, Li H, Sun D, . Cancer screening in China: the current status, challenges, and suggestions. Cancer Lett. 2021;506:120-127. doi:10.1016/j.canlet.2021.02.017 33684533

