# Supplementary material for: Estimated Cost-effectiveness of Endoscopic Screening for Upper Gastrointestinal Tract Cancer in High-Risk Areas in China
Source: JAMA Netw Open. 2021 Aug 17;4(8):e2121403. doi: 10.1001/jamanetworkopen.2021.21403 (PMC8371571; doi:10.1001/jamanetworkopen.2021.21403)
Supplement: Supplement. — eMethods. eFigure 1. Flowchart of the Overall Participants in the Multicenter Randomized Trial Project eFigure 2. Flowchart of the Participants in the High-Risk Areas eFigure 3. Flowchart of the Screening and Reexamination eFigure 4. Markov Model of Upper Gastrointestinal Tract Cancer (Including Esophageal Cancer and Gastric Cancer) Progression eFigure 5. Model Projected Age-Specific GC Incidence and Mortality Rates Compared to 2 Folds of Observed National GC Incidence and Mortality Rates in 2015 eFigure 6. Model Projected Age-Specific EC Incidence and Mortality Rates Compared to 2 Folds of Observed National EC Incidence and Mortality Rates in 2015 eTable 1. Prevalence Rates (%) of EC/GC-Related Health States Used in the Model, by Initial Screening Age eTable 2. Annual Transition Probabilities Used in the Model eTable 3. State-Specific Compliances with Treatment Estimated Based on Our Project eTable 4. Costs of Screening Estimated Based on Our Project eTable 5. Costs of EC/GC-Related Treatment in Different Disease Progression Stages Based on the Survey Included in Our Project (US$) eTable 6. Utility Scores of EC/GC-Related Health States Based on the Survey Included in Our Project eReferences. [file jamanetwopen-e2121403-s001.pdf]

Supplementary Online Content

Xia R, Zeng H, Liu W, et al. Estimated cost-effectiveness of endoscopic screening for upper gastrointestinal tract cancer in high-risk areas in China. *JAMA Netw Open*. 2021;4(8):e2121403. doi:10.1001/jamanetworkopen.2021.21403

eMethods.
eFigure 1. Flowchart of the Overall Participants in the Multicenter Randomized Trial Project
eFigure 2. Flowchart of the Participants in the High-Risk Areas
eFigure 3. Flowchart of the Screening and Reexamination
eFigure 4. Markov Model of Upper Gastrointestinal Tract Cancer (Including Esophageal Cancer and Gastric Cancer) Progression
eFigure 5. Model Projected Age-Specific GC Incidence and Mortality Rates Compared to 2 Folds of Observed National GC Incidence and Mortality Rates in 2015
eFigure 6. Model Projected Age-Specific EC Incidence and Mortality Rates Compared to 2 Folds of Observed National EC Incidence and Mortality Rates in 2015
eTable 1. Prevalence Rates (%) of EC/GC-Related Health States Used in the Model, by Initial Screening Age
eTable 2. Annual Transition Probabilities Used in the Model
eTable 3. State-Specific Compliances with Treatment Estimated Based on Our Project
eTable 4. Costs of Screening Estimated Based on Our Project
eTable 5. Costs of EC/GC-Related Treatment in Different Disease Progression Stages Based on the Survey Included in Our Project (US$)
eTable 6. Utility Scores of EC/GC-Related Health States Based on the Survey Included in Our Project
eReferences.

This supplementary material has been provided by the authors to give readers additional information about their work.

© 2021 Xia R et al. *JAMA Network Open*.

eMethods. Overview of the multicenter randomized controlled trial (RCT) of upper gastrointestinal tract cancer (UGIC) screening in China

Trial design and participants

To further determine the feasibility and efficacy of endoscopic screening for both EC and GC, in May 2015, the National Cancer Center (NCC) of China/Cancer Hospital, Chinese Academy of Medical Sciences (CICAMS) launched a multicenter community-based cluster RCT project in three high-risk areas (Linzhou County of Henan Province, Cixian County of Hebei Province, and Wuwei County of Gansu Province) and four non-high-risk areas (Sheyang County of Jiangsu Province, Luoshan County of Henan Province, Harbin City of Heilongjiang Province, and Changsha City of Hunan Province). The project was registered with the Protocol Registration System in the Chinese Clinical Trial Registry (identifier: ChiCTR-EOR-16008577) and approved by the independent Ethics Committee of the NCC/CICAMS (2015SQ00223). Design details and initial results have been previously published.[1,2]

In total, 345 eligible villages/communities in seven screening centers constituted the randomization unit: 163 units from the three high-risk areas and 182 units from the four non-high-risk areas. Based on a stratified cluster sampling design, these units were randomly allocated to the screening arm or control arm at a ratio of 1:1 by each center for practical reasons and contamination prevention (eFigure 1−2). According to the study group assignment, local village doctors or community public health workers at each site recruited and assigned participants to each group. The eligible participants were residents aged 40–69 years with no personal history of cancer and who did not undergo endoscopy in the past three years.

Screening, reexamination and treatment

The screening and re-examination procedures are shown in eFigure 3. The diagnoses were reported according to the American Joint Committee on Cancer Staging System (7th ed.). Stage I and II tumors were categorized as early cancer, and stage III and IV tumors were categorized as advanced cancer. The participants from the high-risk areas were automatically identified as high-risk individuals and were invited to undergo endoscopic screening. The participants from the non-high-risk areas were evaluated with a risk assessment questionnaire, and only subjects who were identified as high-risk individuals were invited to undergo endoscopic screening. The screened participants were given a local anesthetic, and the entire esophagus and stomach were visually examined. Lugols' iodine staining in the esophagus and indigo carmine dye in the stomach were performed as necessary to aid in the diagnosis of suspicious lesions. Suspicious lesions were targeted for biopsy for further pathological diagnosis. Subjects without suspicious lesions did not undergo a biopsy.

Individuals with precancerous lesions were followed up by endoscopic re-examinations. A triennial endoscopic re-examination was required for mild esophageal dysplasia (mD), and an annual re-examination was required for moderate esophageal dysplasia (MD) or low-grade gastric intraepithelial neoplasia (LGIN).

The corresponding treatment was provided according to the diagnosis results. If the early lesions are histologically confirmed, the participants were recalled to the clinic, and intervention methods appropriate for the lesion severity were employed. For severe esophageal dysplasia/carcinoma in situ (SD/CIS), high-grade gastric intraepithelial neoplasia/carcinoma in situ (HGIN/CIS), early esophageal cancer (EC), or gastric cancer (GC), endoscopic mucosal resection or endoscopic submucosal dissection treatments were used as local therapies. For advanced EC or GC, the therapies included esophagectomy, radical operation, radiotherapy, and other conventional treatments.

© 2021 Xia R et al. *JAMA Network Open*.

eFigure 1. Flowchart of the overall participants in the multicenter randomized trial project.

© 2021 Xia R et al. *JAMA Network Open*.

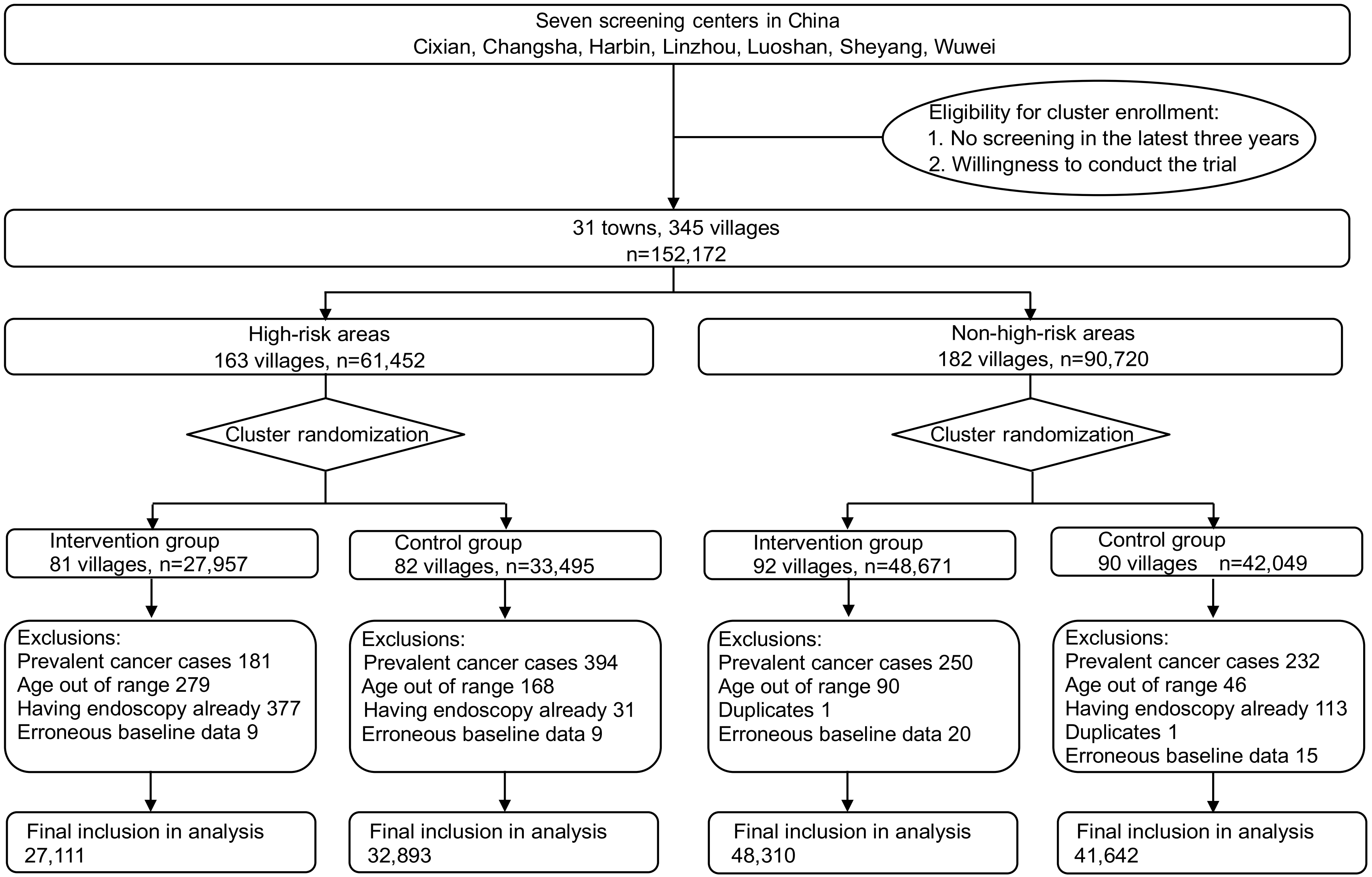

© 2021 Xia R et al. *JAMA Network Open*.

eFigure 2. Flowchart of the participants in the high-risk areas.

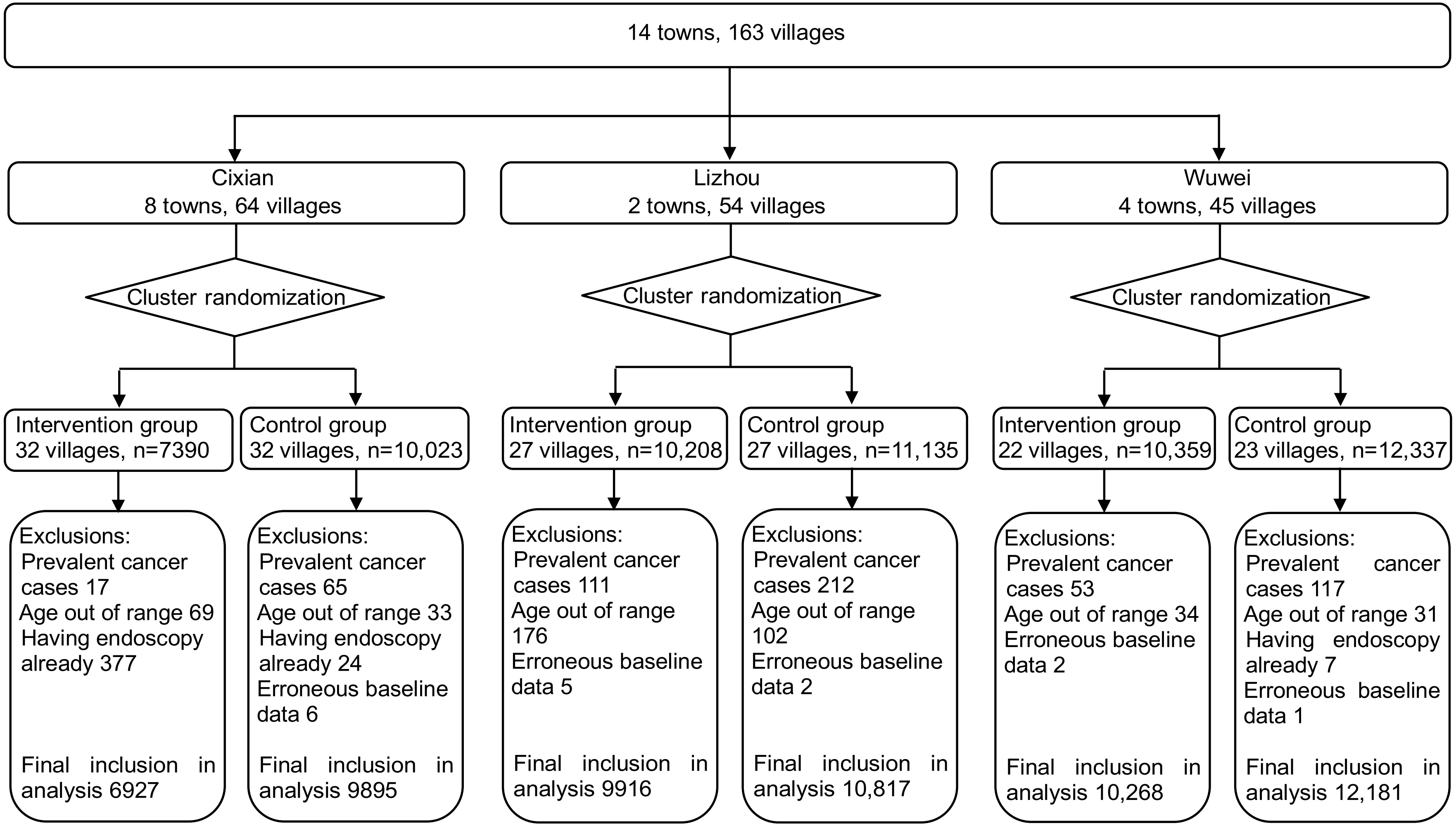

© 2021 Xia R et al. *JAMA Network Open*.

eFigure 3. Flowchart of the screening and reexamination

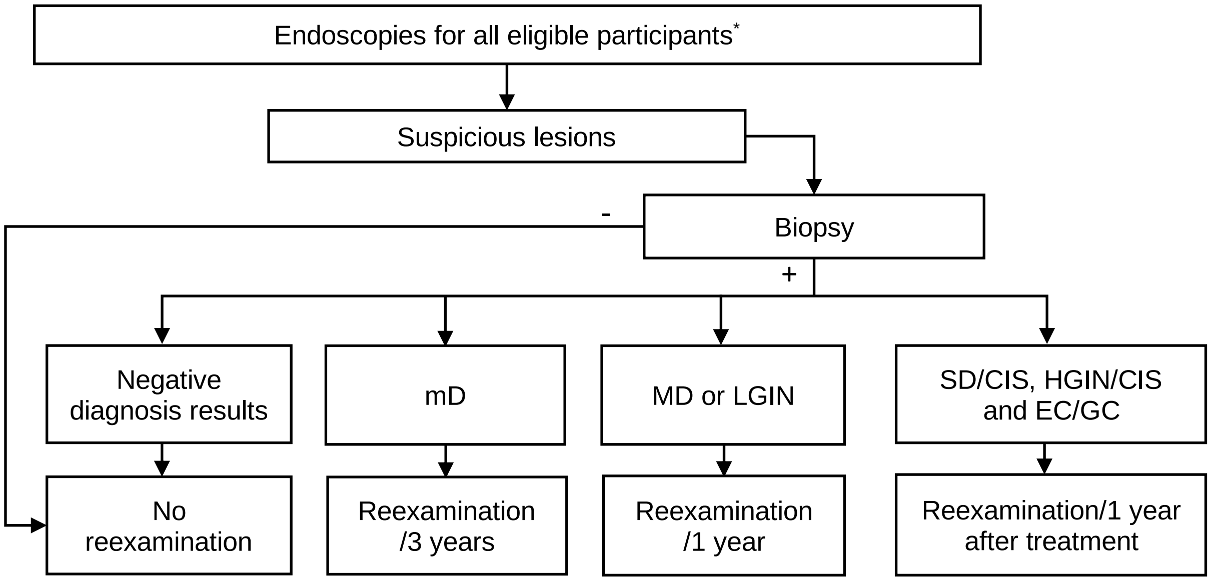

Eligible participants*: All individuals aged 40–69 years from high-risk areas; high-risk individuals aged 40–69 years from non-high-risk areas.
Abbreviations: mD, mild dysplasia; MD, moderate dysplasia; SD/CIS, severe dysplasia/carcinoma in situ; EC, esophageal cancer; LGIN, low-grade intraepithelial neoplasia; HGIN/CIS, high-grade intraepithelial neoplasia/carcinoma in situ; GC, gastric cancer.

2. Markov model

eFigure 4 shows our Markov model of the progression of UGIC, which includes both EC and GC. Circles represent health states, and solid lines with arrowheads represent transitions and their directions. Dotted lines represent persons with precancerous lesions or cancer identified by self-initiated examinations or screening without a time delay of state transition.A person in the detected mD, MD or LGIN state will return to an undetected state if the person fails to comply with the regular re-examination or a false-negative re-examination result is obtained. A person in the PT-SD/CIS or PT-HGIN/CIS state will return to the normal state if the person maintains the current state for more than 10 years. A person in the PT-early EC or GC state or in the PT-advanced EC or GC state will stay in the current state until death if the person maintains the current state for more than 10 years. The death state (not shown here) is the absorbing state of the model, a person in any other states will enter the death state due to age-specific natural background death, and a person in the detected advanced EC or GC state will face cause-specific mortality from EC or GC in addition to a natural background death rate.

Following the screening and re-examination procedures mentioned above, triennial endoscopic re-examination for detected mild esophageal dysplasia (mD) and annual endoscopic re-examination for detected moderate esophageal dysplasia (MD) and low-grade gastric intraepithelial neoplasia (LGIN) were considered in the model.

© 2021 Xia R et al. *JAMA Network Open*.

eFigure 4. Markov model of upper gastrointestinal tract cancer (including esophageal cancer and gastric cancer) progression.

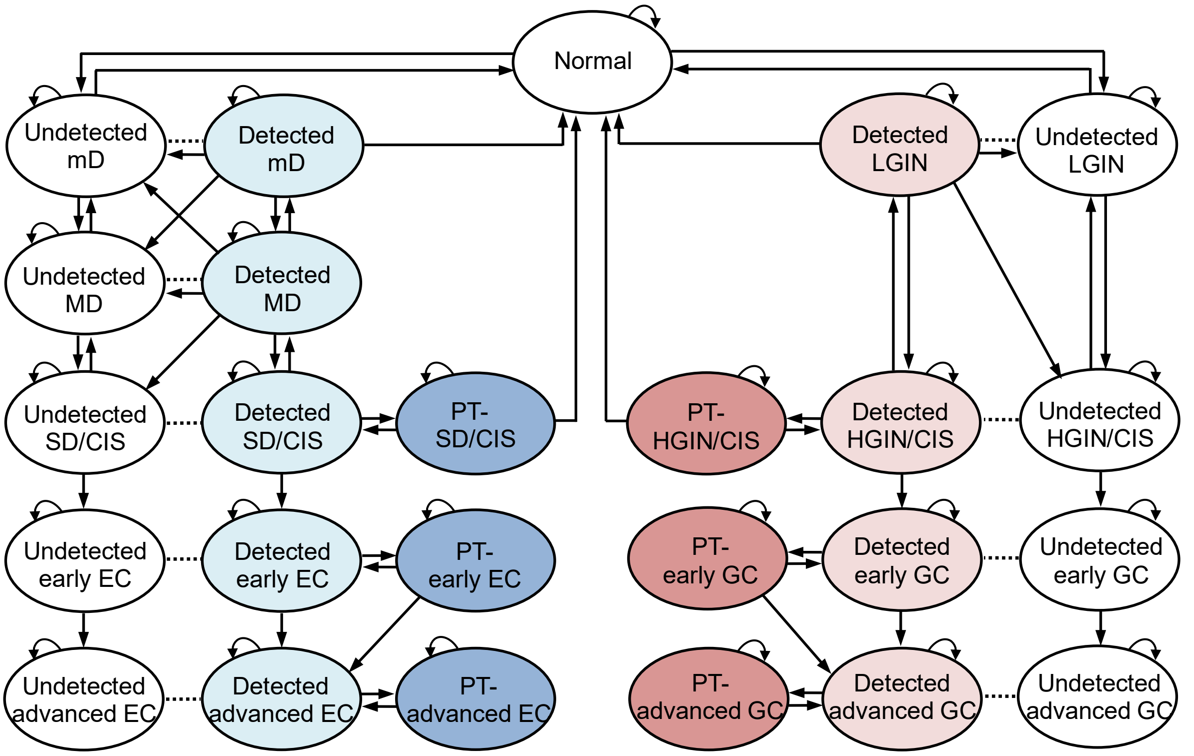

Abbreviations: mD, mild dysplasia; MD, moderate dysplasia; SD/CIS, severe dysplasia/carcinoma in situ; EC, esophageal cancer; LGIN, low-grade intraepithelial neoplasia; HGIN/CIS, high-grade intraepithelial neoplasia/carcinoma in situ; GC, gastric cancer; PT, posttreatment.

3. Model parameters and data sources

Initial probabilities

The initial probabilities of EC/GC-related health states were mainly obtained from screening baseline reports of our project in the high-risk areas. The base-case prevalence rates of EC/GC-related health states were calculated as the proportion of each pathologic stage of EC/GC among the subjects who underwent endoscopy at each initial screening age, which were used to determine the initial distributions of cohort members in health states of the model. Referring to previous reports from China, a wide range was set for each rate to cover the values reported in high-risk areas. Details are presented in eTable 1.

eTable 1. Prevalence rates (%) of EC/GC-related health states used in the model, by initial screening age[a]

		Initial screening age (years)						Reference
		**40−44**	**45−49**	**50−54**	**55−59**	**60−64**	**65−69**	
mD	Base-case value	1.16	2.04	3.39	5.35	8.11	11.90	
	Range	0.58−2.57	1.02−4.52	1.70−7.51	2.68−11.85	4.06−17.97	5.95−26.36	[3–5]
MD	Base-case value	0.10	0.22	0.44	0.83	1.46	2.46	
	Range	0.05−0.27	0.11−0.59	0.22−1.19	0.42−2.24	0.73−3.94	1.23−6.64	[3–5]
SD/CIS	Base-case value	0.05	0.12	0.28	0.58	1.12	2.05	
	Range	0.03−0.16	0.06−0.38	0.14−0.9	0.29−1.86	0.56−3.58	1.03−6.56	[3–5]
Early EC	Base-case value	0.02	0.05	0.11	0.23	0.45	0.85	
	Range	0.01−0.04	0.03−0.10	0.06−0.22	0.12−0.46	0.23−0.90	0.43−1.7	[3–5]
Advanced EC	Base-case value	0.01	0.02	0.04	0.07	0.14	0.25	
	Range	0−0.02	0.01−0.0	0.02−0.08	0.04−0.14	0.07−0.28	0.13−0.5	[3–5]

© 2021 Xia R et al. *JAMA Network Open.*

			4					
LGIN	Base-case value	3.62	4.58	5.65	6.84	8.14	9.55	
	Range	1.81−7.24	2.29−9.16	2.83−11.3	3.42−13.68	4.07−16.28	4.78−19.1	4–6
HGIN/CIS	Base-case value	0.16	0.27	0.44	0.67	1.00	1.43	
	Range	0.08−0.32	0.14−0.54	0.22−0.88	0.34−1.34	0.5−2.00	0.72−2.86	4–6
Early GC	Base-case value	0.06	0.12	0.23	0.42	0.72	1.19	
	Range	0.03−0.12	0.06−0.24	0.12−0.46	0.21−0.84	0.36−1.44	0.60−2.38	4–6
Advanced GC	Base-case value	0.03	0.06	0.11	0.19	0.31	0.50	
	Range	0.02−0.06	0.03−0.12	0.06−0.22	0.10−0.38	0.16−0.62	0.25−1.00	4–6

[a]All the rates are specified as triangular distributions, with the upper and lower limits of range as the minimum and maximum values, respectively, and the base-case value as the most likely value.
Abbreviations: mD, mild dysplasia; MD, moderate dysplasia; SD/CIS, severe dysplasia/carcinoma in situ; EC, esophageal cancer; LGIN, low-grade intraepithelial neoplasia; HGIN/CIS, high-grade intraepithelial neoplasia/carcinoma in situ; GC, gastric cancer.

Annual transition probabilities

The annual transition probabilities were derived from published observational studies concerning the natural history of EC/GC and economic evaluation studies of EC/GC; the probabilities are summarized in eTable 2. It is believed that patients with advanced EC/GC may mainly die from cancer and that patients with early EC/GC or precancerous lesions may not die from cancer. In the model, all posttreatment states were set as tunnel states to control their transition directions, which were associated with the duration of stay in their current states. All parameters were adjusted within a wide range in the sensitivity analyses to cover most of the reported data. In the probabilistic sensitivity analysis, the age-dependent annual transition probabilities were specified as uniform distributions, and the non-age-dependent annual transition probabilities were specified as triangular distributions.

eTable 2. Annual transition probabilities used in the model

Parameter	Base-case value	Range	Distribution	Reference
Normal				
To mD	0.012	±50%	Triangular (0.006, 0.012, 0.018)	7–9
To LGIN	0.007	±50%	Triangular (0.0035, 0.007, 0.0105)	7,10
mD				
To normal	0.05	±50%	Triangular (0.025, 0.05, 0.075)	7–9
To MD	0.05	±50%	Triangular (0.025, 0.05, 0.075)	7–9
MD				
To mD	0.08	±50%	Triangular (0.04, 0.08, 0.12)	7–9
To SD/CIS	0.12	±50%	Triangular (0.06, 0.12, 0.18)	7–9
SD/CIS				
To MD				7–9
40−44 years	0.17	±50%	Uniform (0.085, 0.255)	
45−49 years	0.15	±50%	Uniform (0.075, 0.225)	
50−54 years	0.14	±50%	Uniform (0.07, 0.21)	
55−59 years	0.12	±50%	Uniform (0.06, 0.18)	
60−64 years	0.11	±50%	Uniform (0.055, 0.165)	
≥65 years	0.09	±50%	Uniform (0.045, 0.135)	
To early EC				7–9
40−44 years	0.08	±50%	Uniform (0.04, 0.12)	
45−49 years	0.10	±50%	Uniform (0.05, 0.15)	
50−54 years	0.12	±50%	Uniform (0.06, 0.18)	
55−59 years	0.14	±50%	Uniform (0.07, 0.21)	
60−64 years	0.16	±50%	Uniform (0.08, 0.24)	
≥65 years	0.18	±50%	Uniform (0.09, 0.27)	
Early EC to advanced EC				7–9
40−44 years	0.40	0.25−0.55	Uniform (0.25, 0.55)	

© 2021 Xia R et al. *JAMA Network Open.*

45−49 years	0.45	0.30−0.60	Uniform (0.30, 0.60)	
50−54 years	0.50	0.35−0.65	Uniform (0.35, 0.65)	
55−59 years	0.64	0.49−0.79	Uniform (0.49, 0.79)	
60−64 years	0.71	0.56−0.86	Uniform (0.56, 0.86)	
≥65 years	0.74	0.59−0.89	Uniform (0.59, 0.89)	
Advanced EC to death	0.80	0.58−0.90	Triangular (0.58, 0.80, 0.90)	[7]
LGIN				
To normal	0.04	±50%	Triangular (0.02, 0.04, 0.06)	[6,7,10]
To HGIN/CIS	0.03	±50%	Triangular (0.015, 0.03, 0.045)	[6,7,10]
HGIN/CIS				
To LGIN				[7]
40−44 years	0.17	±50%	Uniform (0.085, 0.255)	
45−49 years	0.15	±50%	Uniform (0.075, 0.225)	
50−54 years	0.14	±50%	Uniform (0.070, 0.210)	
55−59 years	0.12	±50%	Uniform (0.060, 0.180)	
60−64 years	0.11	±50%	Uniform (0.055, 0.165)	
≥65 years	0.09	±50%	Uniform (0.045, 0.135)	
To early GCA				[7]
40−44 years	0.08	±50%	Uniform (0.04, 0.12)	
45−49 years	0.10	±50%	Uniform (0.05, 0.15)	
50−54 years	0.12	±50%	Uniform (0.06, 0.18)	
55−59 years	0.14	±50%	Uniform (0.07, 0.21)	
60−64 years	0.16	±50%	Uniform (0.08, 0.24)	
≥65 years	0.18	±50%	Uniform (0.09, 0.27)	
Early GC to advanced GC				[7]
40−44 years	0.40	0.25−0.55	Uniform (0.25, 0.55)	
45−49 years	0.45	0.30−0.60	Uniform (0.30, 0.60)	
50−54 years	0.50	0.35−0.65	Uniform (0.35, 0.65)	
55−59 years	0.64	0.49−0.79	Uniform (0.49, 0.79)	
60−64 years	0.71	0.56−0.86	Uniform (0.56, 0.86)	
≥65 years	0.74	0.59−0.89	Uniform (0.59, 0.89)	
Advanced GC to death	0.80	0.58−0.9	Triangular (0.58, 0.80, 0.90)	[7]
Recurrence probability after treatment				[7]
PT-SD/CIS to SD/CIS	0.005	±50%	Triangular (0.0025, 0.005, 0.0075)	
PT-early EC				
To early EC	0.05	±50%	Triangular (0.025, 0.05, 0.075)	
To advanced EC	0.10	±50%	Triangular (0.05, 0.10, 0.15)	
PT-advanced EC to advanced EC	0.23	±50%	Triangular (0.115, 0.23, 0.345)	
PT-HGIN/CIS to HGIN/CIS	0.005	±50%	Triangular (0.0025, 0.005, 0.0075)	
PT-early GC				
To early GC	0.05	±50%	Triangular (0.025, 0.05, 0.075)	
To advanced GC	0.10	±50%	Triangular (0.05, 0.10, 0.15)	
PT-advanced GC to advanced GC	0.23	±50%	Triangular (0.115, 0.23, 0.345)	

Abbreviations: mD, mild dysplasia; MD, moderate dysplasia; SD/CIS, severe dysplasia/carcinoma in situ; EC, esophageal cancer; LGIN, low-grade intraepithelial neoplasia; HGIN/CIS, high-grade intraepithelial neoplasia/carcinoma in situ; GC, gastric cancer; PT, posttreatment.

Compliance with treatment

State-specific compliance with treatment was calculated as the proportion of screened patients who actually completed the whole treatment procedure in high-risk areas in our project as summarized in eTable 3. Compliance with treatment in advanced EC patients was assumed to be the same as that in advanced GC patients due to the small and unbalanced number of patients in this disease progression stage in the three study centers. The mean and 95% confidence intervals (CIs) of state-specific compliance with treatment were used as base-case values and ranges in the univariate sensitivity analysis. The beta distributions were calculated using an approximation of the mean and standard deviation (SD) of state-

© 2021 Xia R et al. *JAMA Network Open.*

specific compliance with treatment in the probabilistic sensitivity analysis, with an alpha of $(mean^2) \cdot (1–mean)/(SD^2)$ and a beta of $[mean \cdot (1–mean)/(SD^2) – (mean^2) \cdot (1–mean)/SD^2]$.

eTable 3. State-specific compliances with treatment estimated based on our project

	No. of patients receiving treatment (total No. of patients)				Compliance with treatment	
	Wuwei City	Cixian County	Linzhou County		mean ± SD	95%CI
SD/CIS	30 (35)	44 (62)	58 (80)		0.7458±0.0811	0.5625−0.9654
Early EC	26 (31)	28 (28)	25 (25)		0.9405±0.0931	0.7149−1.0000
Advanced EC[a]	0 (0)	1 (1)	5 (5)		/	/
HGIN/CIS	11 (18)	19 (35)	24 (46)		0.5455±0.0467	0.4425−0.7746
Early GC	50 (62)	23 (24)	53 (54)		0.9000±0.0951	0.6792−1.0000
Advanced GC	10 (11)	7 (7)	10 (10)		0.9643±0.0525	0.8393−1.0000

[a]Compliance with treatment in advanced EC patients was not calculated due to the small and unbalanced number of patients in this disease progression stage in the three study centers.

Abbreviations: SD/CIS, severe dysplasia/carcinoma in situ; EC, esophageal cancer; HGIN/CIS, high-grade intraepithelial neoplasia/carcinoma in situ; GC, gastric cancer; CI, confidence interval; SD, standard deviation.

Costs of screening and EC/GC-related treatment

The cost of screening included screening mobilization and administration costs, endoscopic examination costs, and treatment costs for endoscopic complications, and these data were obtained from the seven study centers in both high-risk and non-high-risk areas in our project.[1,2] The results are shown in eTable 4.

This cost-effectiveness analysis was performed from the healthcare system perspective, and disease state costs in this study only included direct medical costs, including outpatient expenditure, inpatient expenditure, and expenditure for medicines self-purchased in retail pharmacies; however, direct nonmedical costs and indirect costs were not included. The cost of EC/GC-related treatment included the initial treatment cost in the detected states and the subsequent annual healthcare cost in the posttreatment states, which were determined based on the survey administered in our project to assess the economic burden of UGIC in China in 2017.[11] The survey was performed in seven hospitals from seven study centers in six provinces distributed in the eastern, central and western areas of China. In total, 20,105 outpatient records and 20,056 inpatient records were collected to obtain the per-capita outpatient and inpatient expenditures on a single visit/admission, respectively. In total, 2855 patients who were discharged from hospitals for more than one year were interviewed by telephone regarding their medical behaviors in the year of hospitalization and more than one year after discharge, including their outpatient visit rate, inpatient admission rate, self-medication expenditures, etc. Using these data, we calculated the disease stage-specific initial treatment costs and annual healthcare costs as shown in eTable 5.

The point estimates of the costs of screening and EC/GC-related treatment were used as base-case values and allowed ±50% variation in the cost parameters in the univariate sensitivity analysis. We used an approximation of the mean and SD to calculate the distribution in the probabilistic sensitivity analysis as follows: gamma distributions had an alpha of $(mean^2)/(SD^2)$ and a lambda of $mean/(SD^2)$.

© 2021 Xia R et al. *JAMA Network Open.*

eTable 4. Costs of screening estimated based on our project

		Screening mobilization & administration			Endoscopic examination			Treatment for endoscopic complications		
		No. of persons in the screening intervention group	Total cost, US$	Per-capita cost, US$	No. of persons receiving endoscopy examination	Total cost, US$	Per-capita cost, US$	No. of persons with complications in endoscopic examination	Total cost, US$	Per-capita cost, US$
High-risk areas	Wuwei City	10,367	16,848	1.63	8506	416,934	49.02	17	2265	133.24
	Cixian County	10,000	12,277	1.23	9189	483,266	52.59	18	1983	110.17
	Linzhou County[a]	10,061	11,093	1.10	/	/	/	20	1581	79.05
Non-high-risk areas	Changsha City	10,478	9949	0.95	1430	81,591	57.06	3	601	200.33
	Harbin City[b]	/	/	/	421	20,061	47.65	1	150	150
	Sheyang County	10,526	4592	0.44	3035	98,415	32.43	8	1090	136.25
	Luoshan County	12,659	12,770	1.01	3100	129,195	41.68	4	401	100.25
	Total	64,091	67,529	1.05 ± 0.35[c]	25,681	1,229,462	47.87 ± 7.93[c]	71	8071	113.68 ± 36.39[c]

[a]Linzhou County used painless endoscopy for screening, which costs almost twice as much as ordinary endoscopy; thus, it was excluded when calculating this parameter.
[b]Harbin City did not collect the costs of projects for screening mobilization and administration; thus, it was excluded when calculating this parameter.
[c]mean ± standard deviation (SD).

eTable 5. Costs of EC/GC-related treatment in different disease progression stages based on the survey included in our project (US$)

	Outpatient expenditure per visit[a] (a)	Inpatient expenditure per admission (b)	Outpatient visit rate in the year of hospitalization (c)	Inpatient admission rate in the year of hospitalization (d)	Annual outpatient visit rate at more than one year after discharge (e)	Annual self-medication expenditure (f)	Initial treatment cost (mean ± SD) = a × c + b × d	Annual healthcare cost (mean ± SD) = a × e + f
SD/CIS	56	1151	2.13	1.29	1.46	134	1604 ± 879	216 ± 192
Early EC	112	3503	2.42	2.13	1.51	198	7732 ± 5068	367 ± 331

© 2021 Xia R et al. *JAMA Network Open*.

Advanced EC	112	2979	2.58	2.36	1.04	226	7320 ± 4012	342 ± 239
HGIN/CIS	66	1165	1.61	1.13	1.44	148	1423 ± 1200	243 ± 218
Early GC	131	3152	2.52	2.29	1.59	200	7548 ± 3514	409 ± 376
Advanced GC	131	2567	2.76	2.62	1.46	244	7086 ± 2903	435 ± 398

[a]This parameter was assumed to be the same in patients with early and advanced cancers due to the lack of TNM staging information in outpatients.
Abbreviations: SD/CIS, severe dysplasia/carcinoma in situ; EC, esophageal cancer; HGIN/CIS, high-grade intraepithelial neoplasia/carcinoma in situ; GC, gastric cancer; CI, confidence interval; SD, standard deviation.

© 2021 Xia R et al. *JAMA Network Open*.

Utility scores of EC/GC-related health states

Utility scores of EC/GC-related health states were obtained from the survey administered in our project to assess the quality of life of UGIC patients in China.[12,13] The survey was conducted using a case-control design in seven hospitals from seven study centers in six provinces located across the eastern, central, and western regions of China. In total, 2855 UGIC patients and 2179 matched healthy controls completed the Chinese version of the three-level EQ-5D questionnaire by telephone. The EQ-5D was scored using a validated Chinese population-specific value set developed using the time trade-off technique to evaluate the disease stage-specific utility scores as shown in eTable 6.[14] The utility scores ranged from 0 to 1, where 1 represents living without any EC/GC-related disease, and 0 represents death. The mean and 95% CIs of the EQ-5D utility scores were used as base-case values and ranges in the univariate sensitivity analysis. The beta distributions were calculated using an approximation of the mean and SD of the utility scores in the probabilistic sensitivity analysis, with an alpha of $(mean^2) \cdot (1–mean)/(SD^2)$ and a beta of $[mean \cdot (1–mean)/(SD^2) – (mean^2) \cdot (1–mean)/SD^2]$.

eTable 6. Utility scores of EC/GC-related health states based on the survey included in our project

	Sample size[a]	Utility score	
		mean ± SD	95% CI
Controls	2179		
SD/CIS	257	0.84 ± 0.16	0.79−0.89
Early EC	694	0.70 ± 0.21	0.66−0.74
Advanced EC	492	0.61 ± 0.29	0.56−0.66
HGIN/CIS	166	0.92 ± 0.14	0.86−0.99
Early GC	655	0.75 ± 0.19	0.71−0.78
Advanced GC	563	0.57 ± 0.27	0.53−0.62

[a]Excluding 13 EC patients and 15 GC patients with a clinical stage classified as “unknown”.

Abbreviations: SD/CIS, severe dysplasia/carcinoma in situ; EC, esophageal cancer; HGIN/CIS, high-grade intraepithelial neoplasia/carcinoma in situ; GC, gastric cancer; CI, confidence interval; SD, standard deviation.

4. Model validation

In internal validation, seven experts in different fields including clinical, epidemiology, and health economics were invited to confirm the face validity of the model. Two team members independently examined the model programming and calculation results, and gave a unanimous judgment. Model outputs about the tendency of each pathologic grade proportion were checked with the characteristics of natural history of EC/GC. For example, the proportions decreased with the severity of the disease in each age group (mD/LGIN ranked first), and the proportions of each EC/GC pathologic grade increased with age (a maximum in the 80−85 years). We also simulate each parameter change in a broad range to determine whether the direction and magnitude of model outputs behaved as expected.

In external validation, 2 folds of the observed national EC/GC incidence and mortality rates in 2015 were used as references, due to the lack of the reported age-specific data in high-risk areas and the disease characteristics in high-risk areas of China.[15-18] A closed cohort of people aged 40−44 years were assumed to enter the model without screening intervention, and model projected outputs, i.e. projected age-specific EC/GC incidence and mortality rates were compared with the references. The results were shown in eFigure 5−6.

© 2021 Xia R et al. *JAMA Network Open.*

eFigure 5. Model projected age-specific GC incidence and mortality rates compared to 2 folds of observed national GC incidence and mortality rates in 2015.

Abbreviations: EC, esophageal cancer.

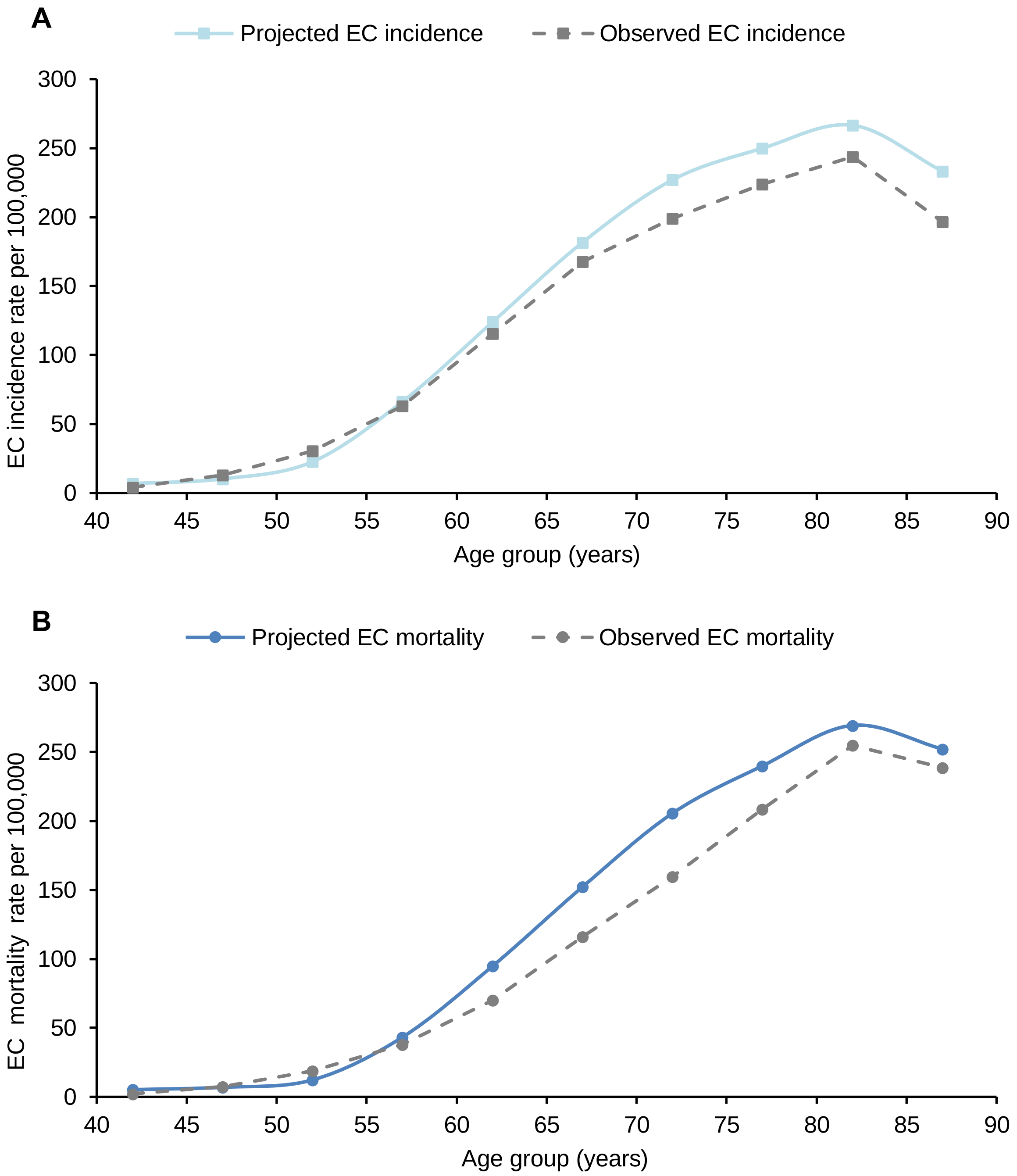

© 2021 Xia R et al. *JAMA Network Open*.

eFigure 6. Model projected age-specific EC incidence and mortality rates compared to 2 folds of observed national EC incidence and mortality rates in 2015.

Abbreviations: GC, gastric cancer.

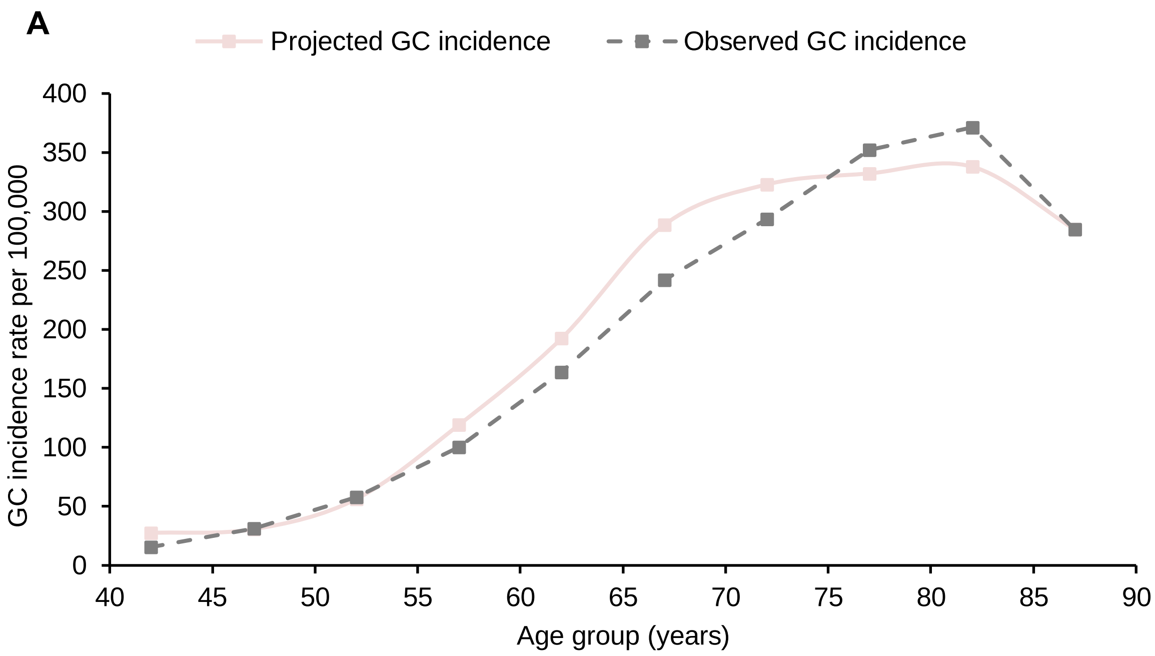

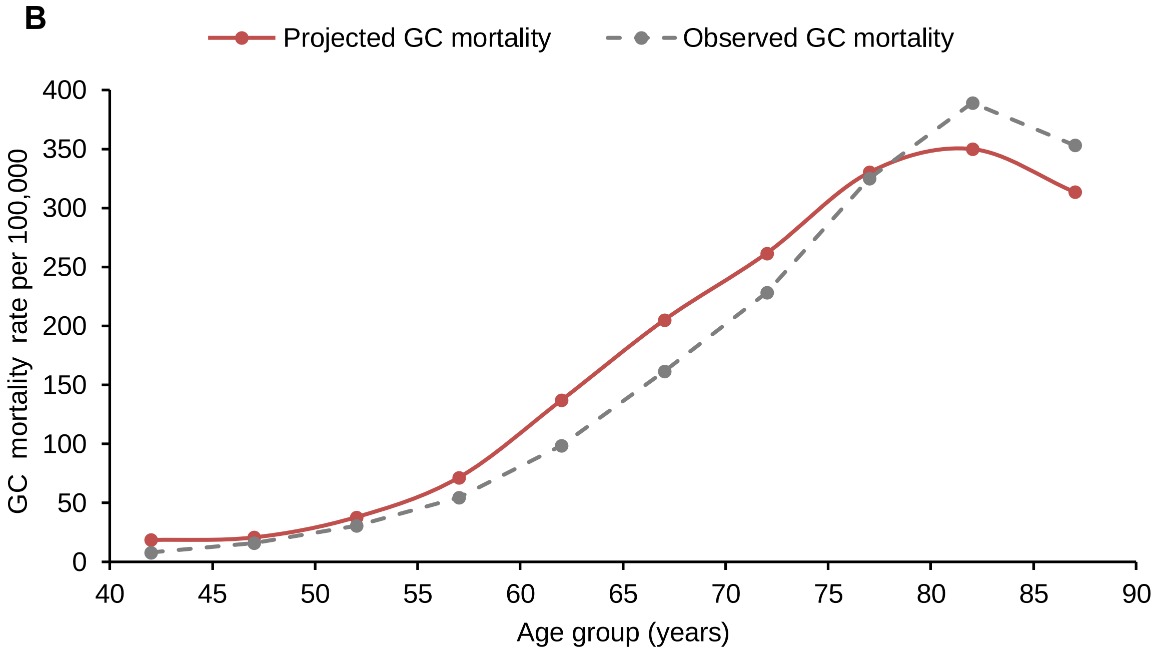

© 2021 Xia R et al. *JAMA Network Open*.

References

1. Chen W, Zeng H, Chen R, et al. Evaluating efficacy of screening for upper gastrointestinal cancer in China: a study protocol for a randomized controlled trial. Chin J Cancer Res. 2017; 29(4):1-9. doi:10.21147/j.issn.1000-9604.2017.04.02

2. Zeng H, Sun K, Cao M, et al. Initial results from a multi-center population-based cluster randomized trial of esophageal and gastric cancer screening in China. BMC Gastroenterol. 2020;20:398-409. doi:10.1186/s12876-020-01517-3

3. Wang M, Hao CQ, Zhao DL, et al. Distribution of esophageal squamous cell cancer and precursor lesions in high-risk areas, Linzhou in Henan province and Feicheng in Shandong province of China, 2005-2009. Chinese Journal of Preventive Medicine. 2015;49(8):677-682.

4. Zheng X, Mao X, Xu K, et al. Massive Endoscopic Screening for Esophageal and Gastric Cancers in a High-Risk Area of China. PLOS ONE. 2015;10(12):e0145097. doi:10.1371/journal.pone.0145097

5. Liang S, Li K, Gong J, et al. Results of the endoscopic screening program of esophageal and gastric cardia cancers using iodine staining in Feicheng, Shandong Province, from 2006 to 2012. Chinese journal of oncology. 2015;37(7):549-552.

6. You WC. Intervention on gastric cancer and precancerous lesions -- the practice of gastric cancer at high-risk area for 23 years. Journal of Peking University(Health Sciences). 2006;38:565-570.

7. Yang J, Wei WQ, Niu J, et al. Cost-benefit analysis of esophageal cancer endoscopic screening in high-risk areas of China. World Journal of Gastroenterology. 2012;18(20):2493-2501. doi:10.3748/wjg.v18.i20.2493

8. Wang GQ, Abnet CC, Shen Q, et al. Histological precursors of oesophageal squamous cell carcinoma: results from a 13 year prospective follow up study in a high-risk population. Gut. 2005;54(2):187-192. doi:10.1136/gut.2004.046631

9. Wang LD, Yang HH, Fan ZM, et al. Cytological screening and 15 years' follow-up (1986-2001) for early esophageal squamous cell carcinoma and precancerous lesions in a high-risk population in Anyang County, Henan Province, Northern China. Cancer Detect Prev. 2005;29:317-322. doi:10.1016/j.cdp.2005.06.004

10. Zhang Y, Zhang L, Pan KF, et al. Prognosis of intestinal metaplasia and expressions of biomarkers in high-risk populations of gastric cancer in Shangdong province. World Chinese Journal of Digestology. 2006;14:2306-2310. doi:10.1160/TH08-01-0056

11. Yang Z, Zeng H, Xia R, et al. Annual cost of illness of stomach and esophageal cancer patients in urban and rural areas in China: A multi-center study. Chin J Cancer Res. 2018;30(4):439-448. doi:CNKI:SUN:ZHAY.0.2018-04-007

12. Liu Q, Zeng H, Xia R, et al. Health-related quality of life of esophageal cancer patients in daily life after treatment: A multicenter cross-sectional study in China. Cancer Medicine. 2018;7(11):5803-5811. doi:10.1002/cam4.1817

13. Xia R, Zeng H, Liu Q, et al. Health-related quality of life and health utility score of patients with gastric cancer: A multi-centre cross-sectional survey in China. Eur J Cancer Care. 2020;29(6):e13283. doi:10.1111/ecc.13283

14. Liu GG, Wu H, Li M, et al. Chinese time trade-off values for EQ-5D health states. Value in Health. 2014;17(5):597-604. doi:10.1016/j.jval.2014.05.007

15. Zhang S, Sun K, Zheng R, et al. Cancer incidence and mortality in China, 2015. Journal of the National Cancer Center, 2020;1(2021):2-11. doi:10.1016/j.jncc.2020.12.001

16. Li JY, Liu BQ, Li GY, et al. Atlas of cancer mortality in the People's Republic of China: an aid for cancer control and research. International journal of epidemiology. 1981;10(2):127-33. doi: 10.1093/ije/10.2.127.

17. Wei WQ, Yang J, Zhang SW, et al. Esophageal cancer mortality trends during the last 30 years in high risk areas in China: comparison of results from national death surveys conducted in the 1970's, 1990's and 2004-2005. Asian Pacific Journal of Cancer Prevention. 2011;12(7):1821-1826.

18. Lin Y, Totsuka Y, Shan B, et al. Esophageal cancer in high-risk areas of China: research progress and challenges. Annals of Epidemiology. 2016;27(3):215-221. doi:10.1016/j.annepidem.2016.11.004

© 2021 Xia R et al. *JAMA Network Open*.
